# Diagnosis of T-cell-mediated kidney rejection by biopsy-based proteomic biomarkers and machine learning

**DOI:** 10.3389/fimmu.2023.1090373

**Published:** 2023-02-06

**Authors:** Fei Fang, Peng Liu, Lei Song, Patrick Wagner, David Bartlett, Liane Ma, Xue Li, M. Amin Rahimian, George Tseng, Parmjeet Randhawa, Kunhong Xiao

**Affiliations:** ^1^ Department of Pharmacology and Chemical Biology, School of Medicine, University of Pittsburgh, Pittsburgh, PA, United States; ^2^ Department of Biostatistics, University of Pittsburgh, Pittsburgh, PA, United States; ^3^ Allegheny Health Network Cancer Institute, Pittsburgh, PA, United States; ^4^ Department of Chemistry, Michigan State University, East Lansing, MI, United States; ^5^ Department of Industrial Engineering, University of Pittsburgh, Pittsburgh, PA, United States; ^6^ Department of Pathology, The Thomas E Starzl Transplantation Institute, University of Pittsburgh, Pittsburgh, PA, United States; ^7^ Center for Proteomics & Artificial Intelligence, Allegheny Health Network Cancer Institute, Pittsburgh, PA, United States; ^8^ Center for Clinical Mass Spectrometry, Allegheny Health Network Cancer Institute, Pittsburgh, PA, United States

**Keywords:** biomarker, quantitative proteomics, machine learning, FFPE, kidney transplantation, diagnosis, mass spectrometry

## Abstract

**Background:**

Biopsy-based diagnosis is essential for maintaining kidney allograft longevity by ensuring prompt treatment for graft complications. Although histologic assessment remains the gold standard, it carries significant limitations such as subjective interpretation, suboptimal reproducibility, and imprecise quantitation of disease burden. It is hoped that molecular diagnostics could enhance the efficiency, accuracy, and reproducibility of traditional histologic methods.

**Methods:**

Quantitative label-free mass spectrometry analysis was performed on a set of formalin-fixed, paraffin-embedded (FFPE) biopsies from kidney transplant patients, including five samples each with diagnosis of T-cell-mediated rejection (TCMR), polyomavirus BK nephropathy (BKPyVN), and stable (STA) kidney function control tissue. Using the differential protein expression result as a classifier, three different machine learning algorithms were tested to build a molecular diagnostic model for TCMR.

**Results:**

The label-free proteomics method yielded 800-1350 proteins that could be quantified with high confidence per sample by single-shot measurements. Among these candidate proteins, 329 and 467 proteins were defined as differentially expressed proteins (DEPs) for TCMR in comparison with STA and BKPyVN, respectively. Comparing the FFPE quantitative proteomics data set obtained in this study using label-free method with a data set we previously reported using isobaric labeling technology, a classifier pool comprised of features from DEPs commonly quantified in both data sets, was generated for TCMR prediction. Leave-one-out cross-validation result demonstrated that the random forest (RF)-based model achieved the best predictive power. In a follow-up blind test using an independent sample set, the RF-based model yields 80% accuracy for TCMR and 100% for STA. When applying the established RF-based model to two public transcriptome datasets, 78.1%-82.9% sensitivity and 58.7%-64.4% specificity was achieved respectively.

**Conclusions:**

This proof-of-principle study demonstrates the clinical feasibility of proteomics profiling for FFPE biopsies using an accurate, efficient, and cost-effective platform integrated of quantitative label-free mass spectrometry analysis with a machine learning-based diagnostic model. It costs less than 10 dollars per test.

## Introduction

In the United States alone, over 200,000 people are now living with functioning kidney transplants, and rejection is the major cause for transplant loss (1, 2). Although short-term graft survival is now excellent (*i.e*., 92% and 83% at 1 and 3 years respectively), the 10-year graft survival rate drops to ~60% due to a spectrum of allograft pathology with a variety of distinct mechanisms and therapeutic options (3). Timely diagnosis of pathology is imperative in preserving allograft longevity and has traditionally been achieved using conventional biopsy-based histologic methods. One major allograft injury mechanism is T-cell-mediated rejection (TCMR), a classic model for T-cell-mediated inflammatory diseases. With contemporary immunosuppression, TCMR is less frequent but remains the dominant early rejection phenotype and the end point in many clinical trials (4).

At present, TCMR is mainly diagnosed using the Banff lesion score *i* (Interstitial inflammation) to evaluate the degree of inflammation in non-scarred areas of cortex. This diagnostic method has significant limitations of being descriptive, non-quantitative, and empirically derived, with significant inter-observer variability (5, 6). These limitations could be avoided with a diagnostic method that evaluates molecular changes in the tissue that preceded morphologic legion development. Such a method would use small tissue samples obtained from biopsies and would ideally be evaluated using molecular markers (RNA, DNA or protein) that could be assayed by a more sensitive, reproducible, and quantitative technology.

As the base of commercially available diagnostic tests, mRNA profiling is available as a test that is marketed as the Molecular Microscope or MMDx^®^ system. It offers prospects of improved disease classification but has several inherent limitations such as high cost of mRNA extraction (7, 8). In addition, it’s easy to miss the core with real disease information since the developed technologies become dependent analysis of a small tissue fragment taken from a longer core sent for routine histology (9, 10).

We have focused on working with proteins extracted from formalin-fixed, paraffin-embedded (FFPE) biopsy specimens to develop such a diagnostic assay. Compared with traditional diagnostic methods, proteomics-based tests have many advantages, including superior specificity, sensitivity, and accuracy as well as being quantitative, high-throughput and low cost. These tests also can simultaneously monitor multiple biomarkers, therefore providing a better understanding of disease pathogenesis and a more systematic evaluation of disease status. Compared with conventional biopsy readings by human observers, biopsy-based proteomic profiling can be a powerful tool to enhance biopsy interpretation, especially when combined with computer modeling to predict outcomes.

Predictive modeling, a method of creating models that can identify the likelihood of disease, has been widely discussed in recent years (11, 12). In predictive modeling, machine learning algorithms employ a variety of statistical, probabilistic and optimization methods to learn from known knowledge and to detect useful patterns from large data sets that rely on labeled training data (13). Whereas a multitude of deep learning-based prediction models for kidney transplant pathology have been developed based on the transcriptomic data sets (14), prediction models incorporating proteomic data have yet to be fully explored.

In our previous work, a Tandem Mass Tag (TMT)-based quantitative proteomic workflow was developed for proteomic profiling of FFPE biopsies (15). However, the TMT-based workflow requires tedious procedures with expensive isobaric reagents that likely preclude its incorporation into routine clinical practice. In the current study, a more cost-efficient and easily manageable workflow using label-free proteomic profiling technology was developed to evaluate kidney allograft injuries. We used samples from patients with TCMR or Polyomavirus BK nephropathy (BKPyVN), which is easily confused with on routine light microscopy (9, 16), and samples from patients without either condition to demonstrate proof-of-concept for developing a clinical-friendly workflow. This system uses label-free proteomic profiling technology and machine learning to correctly differentiate three types of biopsy samples.

## Materials and methods

### Materials

All chemicals used in this study were of analytical grade and purchased from Sigma-Aldrich (St. Louis, MO) unless otherwise stated. LC-MS grade solvents, including water, formic acid (FA), methanol and acetonitrile (ACN) were ordered from Fisher Scientific (Pittsburgh, PA). The 10-kDa centrifugal filter unit was purchased from Millipore Sigma (Burlington, MA). The complete mini protease inhibitor cocktail was from Roche (Indianapolis, IN).

### Patients and sample collection

This study was approved by the University of Pittsburgh IRB (protocol 10110393). STA kidney specimens and biopsies diagnosed as TCMR or BKPyVN were selected from weekly clinical conferences conducted immediately prior to commencement of the study. Diabetes mellitus, hypertension, and glomerulonephritis were the three most common causes of end-stage kidney disease in these subjects. All patients received Thymoglobulin induction followed by dual maintenance immunosuppressive therapy consisting of mycophenolate mofetil and tacrolimus. Corticosteroids were tapered over the first 7 days and then discontinued. Histologic diagnoses were based on the Banff classification for kidney allograft pathology (17). Diagnostically relevant Banff scores for the TCMR patients were g0, v0, i2, ptc0, cg0, ci1, ct1 for all biopsies. The t -score was 2 in all biopsies, except for 1 biopsy in which it was t3. The core needle biopsy specimens (18 gauge) were fixed in formalin immediately and paraffin embedded within 24 h.

The patients (eight males, seven females) in the discovery cohort ranged in age from 32 to 84 years with mean values of 60.8, 56.2, and 51.6 in the STA, BKPyVN, and TCMR groups, respectively. Biopsies had been performed 23-526 days post-transplant (mean 263) and showed renal cortex with mild interstitial fibrosis and tubular atrophy. For the BKPyVN biopsies, the concentration of viral loads ranged from 2.38E+08 to 6.67E+10 copies per mL in the urine and 8.11E+03 to 3.85E+05 copies per mL in the plasma. All biopsies showed polyomavirus antigens on immunohistochemistry.

The patients (two males, seven females) in validation cohort ranged in age from 27 to 73 years with mean values of 52.7. Biopsies had been performed 74-106 days post-transplant (mean 88).

### Deparaffinization and protein extraction

The biopsy tissue embedded in the paraffin blocks was extracted with a sharp scalpel, followed by cutting into 1 mm pieces. Each sample was deparaffinized by incubating with 1 mL of xylene at room temperature (RT) for 5 min, centrifugating at 3,000 × g for 2 min. The supernatant was discarded after centrifugation. The above xylene washing step was repeated three times. The deparaffinized sample was rehydrated by incubating with 1 mL of 100% ethanol at RT for 3 min. The sample was centrifugated at 3,000 × g for 2 min, with the supernatant discarded. The ethanol washing step was repeated three times. After ethanol washing, 40 µL of lysis buffer (2% sodium dodecyl sulfate (SDS), 20 mM tris(hydroxymethyl)aminomethane (Tris), 1% protease inhibitor, pH 8.0) was added to each sample, which was then subjected to a focused ultrasonication step (work 4s, suspend 6s, total time 2min) with Model 120 Sonic Dismembrator (Fisher Scientific, Pittsburgh, PA). After the focused ultrasonication repeated for five times, the disrupted samples were incubated at 98˚C for 120 min. With the supernatants collected by centrifugation at 10,000 × g for 10 min at 4˚C, the concentration of the obtained protein supernatant was measured by BCA Protein Assay Kit (Thermo Scientific, Waltham, MA).

### In-gel digestion

For each FFPE sample, 10 µg of the extracted proteins were respectively subjected to in-gel trypsin digestion according to standard procedures with minor modifications (18, 19). Briefly, the protein concentration was adjusted to 1 mg/mL with lysis buffer. 4 × sample loading buffer was added to a final concentration of 1 x and Tris(2-carboxyethyl) phosphine (TCEP) was added to a final concentration of 10 mM. The protein samples were denatured and reduced by incubating at 90°C for 20 min. After cooling down to the room temperature, the samples were alkylated by incubating with 25 mM IAA at room temperature in the dark for 30 min. The protein samples were then loaded into the wells of an SDS-polyacrylamide gel electrophoresis (SDS-PAGE) gel (i.e., 4% stacking gel and 10% separating gel). The gel electrophoresis was stopped once the dye front migrated into the separating gel and reached about 1cm from the top of the separating gel. After Coomassie blue staining, the 1 cm long gel band corresponding to proteins were excised and chopped into about 20 small pieces. The gel pieces were distained by incubating with 50% ACN in 50 mM ammonium bicarbonate (NH_4_HCO_3_) for 15 min at 37°C with sharking for three times, followed by incubating with pure water for 1 h at 37°C with shaking for three times. Subsequently, the gel pieces were treated with 100% ACN, followed by rehydration with digestion buffer (50 mM NH_4_HCO_3_ buffer containing 2% ACN). Protein tryptic digestion was performed at 37°C for overnight with 10 ng/µl trypsin (Promega, Mannheim, Germany). Digestion was terminated with FA at a concentration of 1% (v/v). Finally, the tryptic digests were extracted by incubating with 50% ACN followed by 80% ACN, purified with stage-tip protocol (20) and lyophilized with a vacuum concentrator (Thermo Scientific, Waltham, MA).

### On-filter digestion

For each FFPE sample, 10 µg of the extracted proteins were processed using a filter-aided sample preparation (FASP) method (21) with minor modifications. Briefly, the proteins were denatured and reduced by incubating with 100 mM TCEP for 10 min at 90°C. After the sample was cooled down to RT, 100 µL of 8 M urea (dissolved in 100 mM NH_4_HCO_3_) was added to the sample and mixed. Then the mixture of each sample was loaded onto a 10-kDa centrifugal filter unit (250 µL/unit) followed by centrifugation at 14,000 g for 20 min. After the proteins on the membrane were washed with 200 µL of 8 M urea once, 200 µL of 8 M urea containing 20 mM IAA was added to the membrane and incubated at RT for 30 min in the dark. Next, the proteins on the membrane were washed with 200 µL of 8 M urea three times followed by 100 mM NH_4_HCO_3_ three times. Finally, 150 µL of 100 mM NH_4_HCO_3_ (pH 8.0) containing 0.4 µg of trypsin was added to each unit and incubated at 37°C for overnight. After digestion, FA was used to acidify the protein digests to terminate digestion at 1% FA (v/v). The filtrate units were centrifuged at 14,000 g for 15 min, and the flow-through containing the peptides was collected. To increase peptide recovery from the membrane, the membrane was further washed with 150 µL of water, with elutes lyophilized with a vacuum concentrator.

### Liquid chromatography with tandem mass spectrometry

The LC-MS/MS experiments were performed using nanoACQUITY Ultra-Performance LC (UPLC) system (Waters, Milford, MA) coupled with a LTQ Orbitrap Velos mass spectrometer (Thermo Scientific, San Jose, CA). Peptide separation was performed on a C18 capillary column (10.5 cm, 3 μm, 120 Å) from New Objective (Woburn, MO). The two eluent buffers were H_2_O with 2% ACN and 0.1% FA (mobile phase A), and ACN with 2% H_2_O and 0.1% FA (mobile phase B), and both were at pH 3. The gradient of the mobile phase B was set as follows: sample loaded at 2% B for 10 min, then 2%-35% B in 45 min, 35%-98% B in 10 min, and maintained at 80% B for 10 min. After separation, the column was equilibrated at 2% B for 25 min. The flow rate was 350 nL/min.

The LTQ Orbitrap Velos mass spectrometer was operated in the data-dependent acquisition (DDA) mode. MS1 scans were acquired in the Orbitrap analyzer at a resolution of 1.5 × 10^4^ over the *m/z* 350-1,500 range. The AGC targets were set as 1 × 10^6^ and 5 × 10^3^ for MS scans and MS/MS scans, respectively. The ion accumulation times were set as 60 ms for MS scans and 50 ms for MS/MS scans. To improve spectrum utility, only ions with charge state between 2 and 4 were subjected to fragmentation with a minimum signal threshold of 500. The 20 most intense ions were fragmented at a normalized collision energy of 35%. Tandem mass spectra were acquired in the ion trap. The dynamic exclusion time was set to 30 s, with the isolation window as 2 Da. For MS2, the selected precursor ions were fragmented with activation time of 20 ms while activation q as 0.25.

### Data analysis

Raw data files were processed using Proteome Discoverer (Thermo Scientific, version 1.4) with SEQUEST search engine. MS/MS spectra were matched with a Uniprot *Homo sapiens* database (204,961 entries, May 2022) and *BK polyomavirus (strain AS)* (BKPyVN) database (5 entries, May 2022), using the following parameters: full trypsin digest with maximum 2 missed cleavages, static modification carbamidomethylation of cysteine (+57.021 Da), dynamic modification of phosphorylation at serine, threonine, or tyrosine (+79.966 Da) as well as oxidation at methionine (+15.995 Da). Precursor and fragment ion mass tolerance was 10 ppm and 0.8 Da, respectively. Peptide spectral matches were validated using percolator with 1% false discovery rate (FDR). To enable meaningful expression comparison of different proteins. The data across different phenotypes were quantile normalized with normalyzer software (22), which was implemented in R using Bioconductor packages. Principal component analysis (PCA) was performed by subjecting data to Perseus software (version 1.6.10.50, available online: https://maxquant.net/perseus/) (23) based on singular value decomposition (24).

### Statistical analysis and bioinformatics analysis

Statistical analysis was performed using the empirical Bayes method implemented in R package LIMMA (25) to determine proteins with statistically significant difference in abundance across different biopsies. To minimize the inaccuracy issues associated with label-free quantitative proteomics, log transformation followed by quantile normalization were performed before quantification analysis (26). DEPs were selected using two criteria: 1) their expression levels in TCMR biopsies significantly changed (*i.e.*, the Benjamin–Hochberg procedure adjusted p value < 0.05) in comparison with STA samples; 2) fold changes (FC) of protein expression levels between TCMR and STA are >2 or <0.5.

Bioinformatics analysis, including protein localization and signaling pathways involved by the identified proteins, was performed using QIAGEN Ingenuity Pathway Analysis (IPA) (https://digitalinsights.qiagen.com). Database for Annotation, Visualization and Integrated Discovery (DAVID) (https://david.ncifcrf.gov/) was performed for the functional annotation of the identified proteins (27, 28). In addition, the protein-protein interaction was predicted using the STRING software (https://string-db.org/) with a confidence cutoff as 0.7, followed by visualization using Cytoscape software (https://cytoscape.org/).

### Machine learning and validation

Three machine learning predictive models were used: linear discriminant analysis (LDA), support vector machine (SVM), and random forest (RF). LDA uses Gaussian assumptions and Bayes theorem to estimate the posterior probability of being classified as TCMR for each testing sample (29). Those with posterior probabilities greater than or equal to a specific cutoff are classified as TCMR. LDA was implemented by the “lda” function in the R package “MASS”. The second method SVM separates the STA and TCMR samples by finding a higher-dimension hyperplane that maximizes the margin, which is the minimum distance of the objects to the hyperplane (30). SVM was implemented by the “svm” function in the R package “e1071”. RF classifies the samples by a majority vote of random trees using the classification and regression tree algorithm. The trees are constructed by bootstrapping of samples and subsampling of features (31). This method was implemented using “randomForest” function in the R package “randomForest”. To evaluate the prediction performance of the protein signatures panel to distinguish TCMR, STA and BKPyVN, we performed a leave-one-out cross-validation (32) and employed the three, above-mentioned learning algorithms (i.e. LDA, SVM and RF) respectively. In each leave-one-out cross-validation procedure, one sample was held out as testing sample and the remaining samples are used as training set (33–35). Missing values were imputed. DEP analysis for the TMT and label-free training sets with all protein features was performed using an empirical Bayes method by R package LIMMA (25). The co-differentially expressed protein (cDEP) features of TMT and label-free proteomics data were then selected. The label-free intensities of selected proteins were used to construct classifiers by implementing the three machine learning algorithms. Then we predict the classification of the testing sample using the classifiers we constructed. Performance was evaluated by calculating sensitivity, specificity, and accuracy. The detailed machine learning code was described in **Supplementary Materials**.

## Results

### Development of a label-free quantitative proteomics workflow for kidney FFPE biopsies

**Figure 1:**
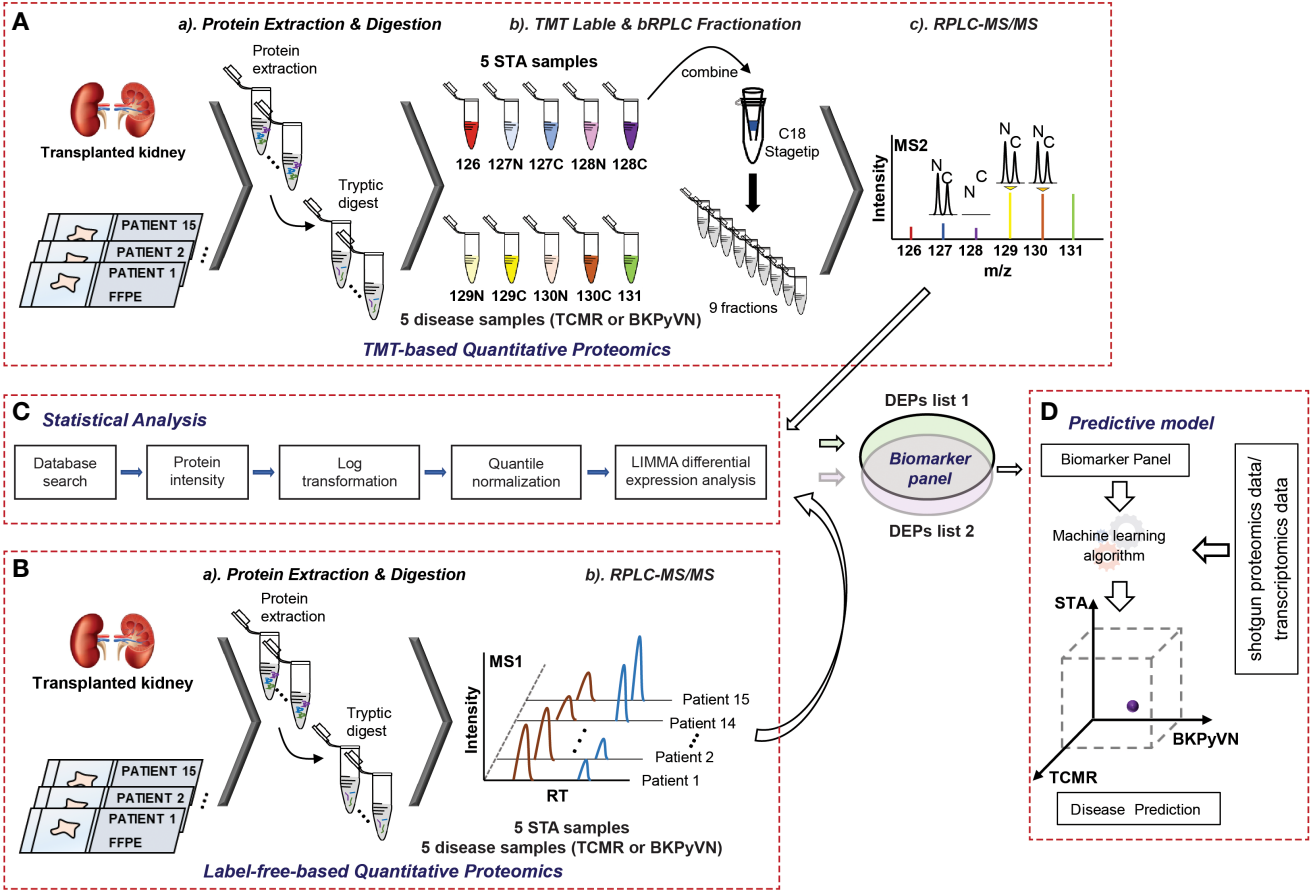
A flow chart showing the procedures to diagnose TCMR by FFPE biopsy-based proteomics and machine learning. **(A)** Experimental procedures for TMT-based quantitative proteomics. The proteins were extracted from 5 TCMR, 5 BKPyVN, and 5 STA biopsies, the digested peptides were labeled with TMT10-plex-reagents and separated by basic reverse phase C18 material. The fractionated peptides were subjected to LC-MS/MS analysis; **(B)** Experimental procedures for label-free quantitative proteomics. The proteins were extracted from 5 TCMR, 5 BKPyVN, and 5 STA biopsies, the digested peptides were directly subjected to LC-MS/MS analysis; **(C)** The proteins were subjected to a systematic statistical analysis consisting of log transformation, quantile normalization, and LIMMA analysis to obtain differentially expressed proteins; and **(D)** The machine learning algorithm was established based on the training data and validated with testing data.

We previously reported a quantitative proteomic workflow for FFPE specimens, consisting of a loss-less sample preparation method, a TMT10plex-proteomic protocol, and a systematic data analysis pipeline (**Figure 1A**) (15). In this work, to simplify the workflow, a modified FASP method, which showed a comparable performance with in-gel digestion strategy for biopsy specimens (data not shown here), was applied for the protein digestion. In addition, instead of labeling the tryptic peptides with isobaric reagents followed by fractionation, the tryptic digests of FFPE specimens were injected directly to the separation column for LC-MS/MS analysis without peptide fractionation (**Figure 1B**). After database search, the identified and quantified proteins were subjected to systematic statistical analysis using the bioinformatics tool of R package LIMMA to obtain DEPs (**Figure 1C**) before building a predictive model (**Figure 1D**). Using this workflow, we analyzed 15 FFPE biopsies containing 5 TCMR, 5 BKPyVN and 5 STA. A total of 800-1350 proteins were identified and quantified with high confidence in each individual sample (**Supplementary Table S1**–**S3**) using a 45 min LC gradient.

### Label-free quantitative proteomics analysis distinguishes different kidney transplant injury biopsies

**Figure 2:**
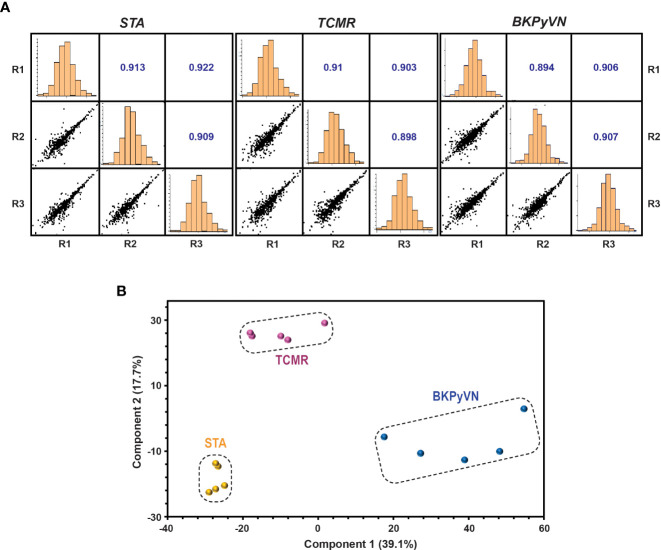
Quantitative proteomic profiling of FFPE biopsies segregates different kidney transplant injuries. **(A)** Repeatability of label-free quantitative analysis. Correlations among 3 replicates for each sample were shown. The correlation coefficient shown in the figure represents the statistical relationship between every two replicates. The larger the value, the higher repeatability between the two replicates. **(B)** A PCA plot obtained by Perseus software demonstrated that the quantified FFPE biopsy proteins were able to segregate TCMR, BKPyVN and STA samples. The PC1 axis is the first principal direction along which the samples show the largest variation. The PC2 axis is the second most important direction, and it is orthogonal to the PC1 axis.

Each step in the label-free proteomics workflow was optimized for FFPE biopsies to improve reproducibility. As shown in **Figure 2A**, a Pearson’s correlation coefficient as high as 0.9 among the replicate experiments was achieved using our label-free workflow, demonstrating a good reproducibility in analyzing FFPE biopsies. To test whether label-free proteomics could distinguish different kidney transplant pathologies from one another, PCA was performed to the label-free proteomic data (**Supplementary Table S4**). As shown in **Figure 2B**, the quantified FFPE proteins not only segregate TCMR biopsies from control specimens (TCMR *vs*. STA), but also distinguish the two tested disease phenotypes from each other (TCMR *vs.* BKPyVN).

### Differential expression analysis reveals potential biomarkers for TCMR

To identify DEPs that can serve as biomarkers to distinguish TCMR from BKPyVN and STA, differential expression analysis was performed using an empirical Bayes method implemented in R package LIMMA (25). In total, 329 out of the 924 quantified proteins were identified as DEPs for TCMR when comparing to STA (**Supplementary Table S5**), with the expression levels of 86 proteins upregulated and 243 downregulated. Similarly, LIMMA analysis revealed that a total of 645 DEPs significantly dysregulated in BKPyVN in comparison to STA biopsies (**Supplementary Table S5**), with the expression levels of 357 proteins upregulated and 288 downregulated. In addition, significant changes in expression levels of 467 proteins in TCMR occurred in comparison with BKPyVN biopsies (**Supplementary Table S5**).

### Build protein signature panels for STA, TCMR and BKPyVN

To build specific protein signature panels for FFPE biopsies of different diseases, the cDEPs that were confidently quantified with the same trend (increase or decrease) in both label-free- (**Supplementary Table S5**) and TMT-based proteomics analyses (**Supplementary Table S6**) were extracted. The STA samples were applied as negative controls for the disease samples. As a result, 106, 40, and 154 proteins were identified as cDEPs in both quantitative proteomics methods for TCMR *vs*. STA, TCMR *vs.* BKPyVN, and BKPyVN *vs.* STA, respectively (**Supplementary Tables S7**and **S8**). As shown in the reference sections in **Supplementary Tables S9** and **S10**, a number of these potential biomarkers were previously reported to be associated with kidney transplant injuries.

### Comparison of different machine learning algorithms for construction of a prediction model for TCMR

**Figure 3:**
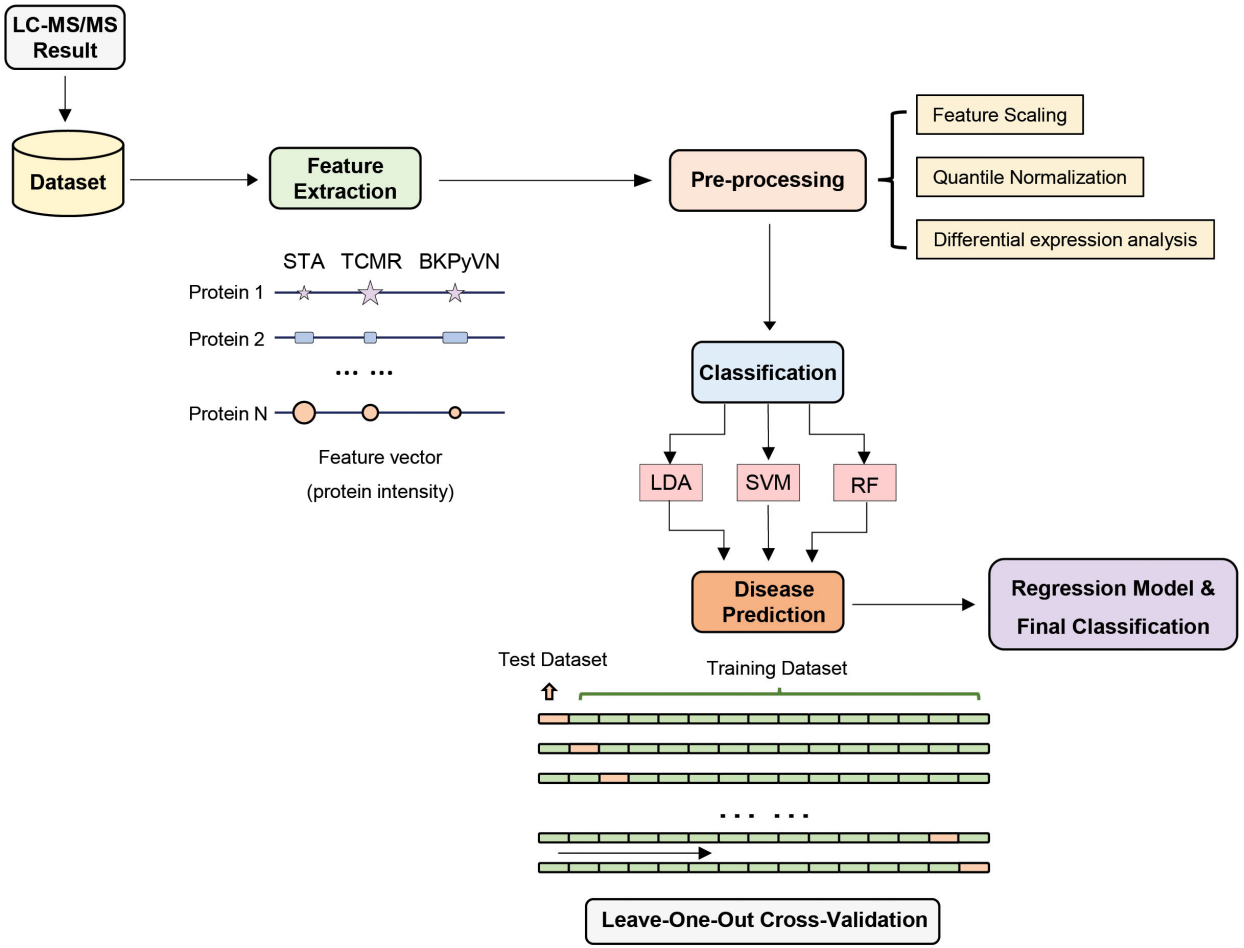
Development of machine learning derived disease prediction model for TCMR, BKPyVN and STA. With the LC-MS/MS data sets of the total of 15 kidney transplant FFPE samples collected, the protein names and corresponding intensities were obtained after database search. Feature selection process selects the critical features (e.g., intensity) for the prediction of kidney rejection disease. After feature selection, preprocessing procedures such as outlier removal, feature scaling (log transformation) and quantile normalization were performed. Various classification techniques were applied to the preprocessed data, with performance evaluated *via* leave-one-out cross-validation strategy. For a total of 15 samples (5 TCMR + 5 BKPyVN + 5 STA), 14 were used as training dataset and the other one as test dataset. Finally, the optimized biomarker panel and disease prediction model were obtained.

**Figure 4:**
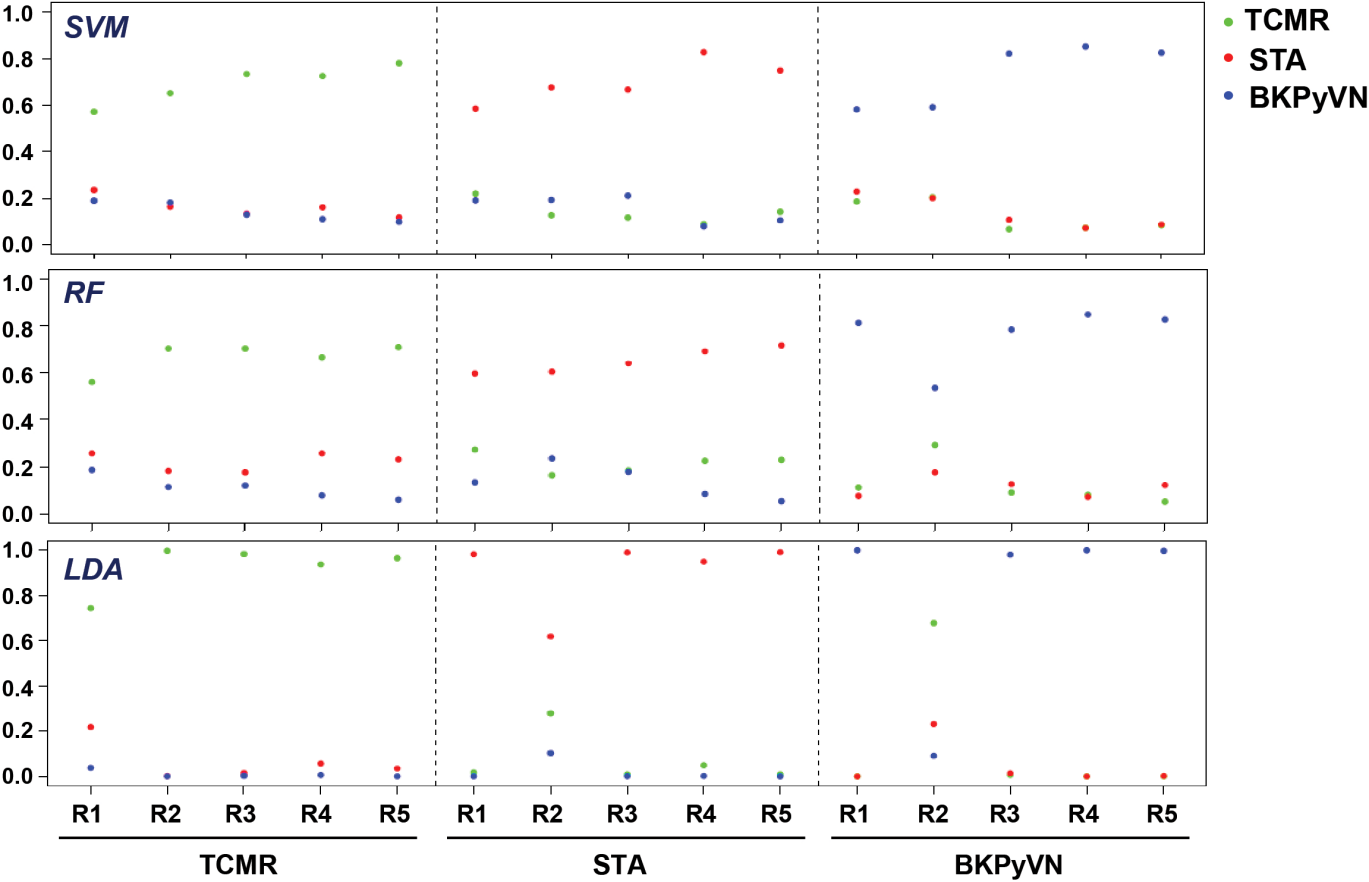
Diagnostic ability of the three different predictive models applied to disease and normal phenotypes. The probability calculated for the kidney transplant biopsy specimens using biomarker panel with the three different prediction models. LDA directly provides posterior probabilities. For random forest (RF), the probabilities are the proportions of votes among the ensembled trees. For SVM, we fit logistic distribution and obtain posterior probabilities by setting probabilities=TRUE in svm function of “e1071” package. R1-R5 are individual samples in each kidney pathology.

To develop a prediction model that can diagnose TCMR, the DEPs commonly quantified from both label-free- and TMT-based quantitative proteomics (**Supplementary Table S7**) were used as the classifiers. After combining the above 106, 40 and 154 cDEPs and removing the overlapped proteins, a total of 247 proteins formed a panel of protein classifiers (**Supplementary Table S11**) for predictive model construction. The detailed procedures to construct the predictive model are outlined in **Figure 3**. Three different machine learning algorithms, *i.e.*, LDA, SVM and RF, were applied to the panel of protein classifiers, respectively. The performance of these machine learning algorithms was compared by using a leave-one-out cross-validation method. During this analysis, each algorithm was performed once for every instance, with the selected instance as a single-item test set and all the other instances as training data set. As shown in **Figure 4**, the disease and normal phenotypes could be obviously distinguished from each other using the three prediction models, with 100%, 100% and 93.3% accuracy achieved in pairwise cross-validation for SVM, RF and LDA, respectively. The receiver operating characteristic (ROC) curve, which has been widely used in clinical epidemiology, was also performed to evaluate the accuracy of our prediction model to discriminate between “diseased” and “non-diseased” (36, 37). For all three algorithms, the area under the curve (AUC) of 1 for the injury subtype provides 100% specificity and 100% sensitivity between each two disease types (**Figure S1**).

### Validation of the TCMR prediction model with blindly tested biopsies

To verify the predictive power of the TCMR prediction models, an independent set of validation samples consisting of 5 TCMR and 5 STA biopsies was used for blind testing. The samples were subjected to label-free proteomics analysis, and the data obtained from each sample was quantile normalized based on the testing data one by one (**Supplementary Tables S12** and **S13**). With the RF-based model, all (100%) the 5 STA and 4 (80%) out of 5 TCMR samples were correctly predicted (80% sensitivity and 100% specificity). With the SVM-based model, 3 (60%) out 5 STA and 4 (80%) out of 5 TCMR samples were correctly predicted (80% sensitivity and 60% specificity). Meanwhile, with the LDA-based model, all (100%) of the 5 STA and 3 (60%) out of the 5 TCMR samples were correctly predicted (60% sensitivity and 100% specificity).

### Validation of the TCMR prediction model using published transcriptome data sets

To further validate the predictive power of the TCMR prediction models, published transcriptome data was used. The classifiers using the 247 proteins from proteomics analysis were applied to two microarray-based data sets [GSE48581 (38) and GSE36059 (39)] posted on the Gene Expression Omnibus website. Applying the three predictive models to GSE36059 achieves 26/35 = 74.3% (SVM), 29/35 = 82.9% (RF) and 25/35 = 71.4% (LDA) in sensitivity as well as 170/281 = 60.5% (SVM), 165/281 = 58.7% (RF) and 182/281 = 64.8% (LDA) in specificity, respectively. Meanwhile, when applied to GSE48581, the sensitivities of the three models are 24/32 = 75% (SVM), 25/32 = 78.1% (RF) and 24/32 = 75% (LDA) and the specificities are 142/222 = 64.0% (SVM), 143/222 = 64.4% (RF) and 136/222 = 61.3% (LDA), respectively.

## Discussion

The FFPE specimen is an invaluable archive for the development of novel molecular diagnostic tests (40). Nucleic acid-based tests have been explored using material from fresh frozen specimens but have been hampered by the low quality and efficiency and high cost of DNA/RNA extraction, along with the scant amount of tissue generally available from needle biopsies (41). We have focused on developing proteomics-based molecular diagnostic tests using proteins extracted from FFPE biopsy specimens.

In comparison with urine and blood, which are also valuable sources in clinical proteomics for disease screening, diagnosis and management as they can be obtained non-invasively, FFPE specimens are advantageous in several aspects. For instance, the FFPE samples are stable at room temperature, and the storage time (up to 32 years) does not have a significant effect on protein identifications from FFPE kidney tissues (42). By contrast, the protein abundance would change significantly when urine was stored up to 3 days at 4°C or up to 6 hours at room temperature (43). Similar changes in protein abundance were observed in blood samples when were stored for 1 month at temperatures above -20°C (44). In addition, the extremely wide concentration range, spanning at least nine orders of magnitude, raises a significant challenge for the discovery of blood biomarkers (45). Due to the differences in the daily intake of fluid, the protein and peptide concentrations widely vary with time of collection in urine samples (43), which limit the study of urine biomarkers. Therefore, FFPE specimens constitute a major part of most archival biobank and provide an invaluable resource for retrospective studies. As biopsy-based histopathologic examination remains essential for evaluating kidney allograft dysfunction, developing clinical proteomics assays using FFPE biopsy specimens is of great significance to assist the pathologists to enhance biopsy interpretation.

In our previous work, a TMT-based quantitative proteomic workflow was developed for molecular profiling of FFPE specimens (15). However, this workflow is not easily manageable in clinical practice for several reasons. First, TMT-labeling reagents are expensive and analyzing TMT-labeled samples requires high-end mass spectrometers with high resolution. Second, the TMT labeling procedures are labor-intensive, and quantitative accuracy is hampered by low labeling efficiency if the experiments are not performed under optimal conditions. Therefore, in this work, we developed a label-free quantitative proteomic workflow for FFPE biopsies as a widely applicable, user-friendly clinical tool, combining the advantages of a simplified sample preparation process with the possibility to perform comparative quantification across many samples. In addition, the cost for reagents can be as low as a few dollars per test.

As a proof-of-principle study, we applied this label-free quantitative proteomic workflow to a small discovery cohort of 15 FFPE biopsies including 5 TCMR, 5 BKPyVN and 5 STA. The high Pearson’s correlation coefficient between the replicate experiments demonstrated that a good reproducibility can be achieved. Although only about one third of proteins (800-1350 proteins) were identified and quantified in the label-free proteomics workflow compared to those (2,798 proteins) in the TMT-based workflow, the PCA clustering result revealed that the obtained label-free proteomic data sets is capable of differentiation among different graft pathologies.

**Figure 5:**
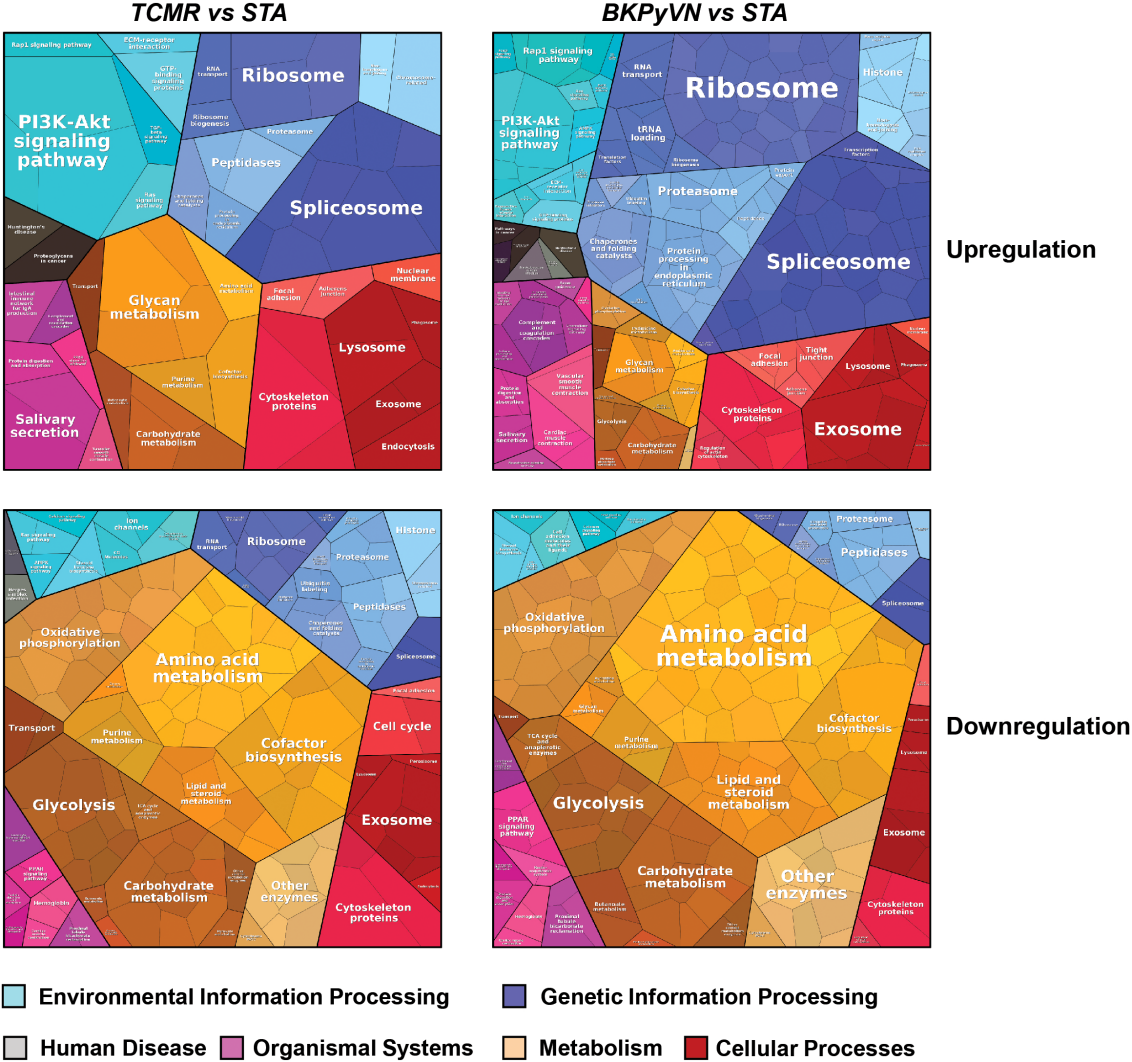
Biological classification of DEPs from label-free quantitative proteome data sets. Treemap of cellular categories altered in disease biopsies in comparison with STA sample illustrated by Proteomaps. The conditions of each disease are marked on the upper side.

To gain insight into disease mechanism, the 329 DEPs between TCMR *vs*. STA and the 645 DEPs between BKPyVN *vs*. STA obtained by label-free proteomics were subjected to bioinformatic analysis. The Proteomap analysis revealed that these two pools of DEPs shared many enriched biological functions and pathways (46). As shown in **Figure 5**, proteins involved in splicing (spliceosome), protein synthesis (ribosome), and PI3K-AKT signaling pathway were enriched in the DEPs upregulated in both TCMR and BKPyVN. Proteins involved in metabolism pathways (*e.g.*, amino acid metabolism, carbohydrate metabolism, lipid and steroid metabolism) and energy production (*e.g.*, oxidative phosphorylation and glycolysis) are mostly downregulated in both disease phenotypes. It is worth mentioning that a number of proteins in the mitochondrial electron transfer chain, responsible for oxidative phosphorylation and ATP synthesis, were downregulated in both TCMR and BKPyVN. Whether the downregulation of the mitochondrial electron transfer chain proteins is one of the causes or the results of the kidney allograft rejection needs to be further investigated.

A similar observation was noted when the 329 DEPs between TCMR *vs*. STA were subjected to STRING analysis (**Figure S2A**). Many proteins involved in electron transfer chain and energy production, for example, oxidoreductases and proteins for glycolysis and oxidative phosphorylation, were expressed at lower levels in TCMR biopsies in comparison with STA specimens. To get more clues to disease mechanism for TCMR, the 329 DEPs between TCMR *vs*. STA were subjected to DAVID analysis; and several groups of proteins stood out (**Supplementary Table S14** and **Figures S2B–E**). Most of these proteins are involved in innate immune system and inflammation response. The first group is collagens (**Figure S2B**). Compared with STA specimens, the expression of the many collagens, including COL1A2, COL6A1, COL6A3, COL1A1, COL4A2, COL6A2 AND COL18A1, was upregulated in TCMR. It is interesting that a recent genome study on the adaptive immune landscape of kidney allograft biopsies showed a significant increase in both formation and degradation of collagens in TCMR compared with STA biopsies (47). The second group of proteins that stood out in the 329 DEPs between TCMR *vs*. STA is the ion channel proteins and transporters. Abnormal ion transport is known to be associated with local or systemic inflammatory response (48). In this study, most of the ion channel proteins and transporters were downregulated in TCMR in comparison with STA (**Figure S2C**). For example, chloride intracellular channel protein CLIC1, which was reported to participate in the regulation of the NLRP3 inflammasome (49), was found to be decreased in its expression in TCMR in comparison with STA. The third group of proteins is protein kinases (**Figure S2D**). As an important class of intracellular enzymes that play a crucial role in most signal transduction cascades, from controlling cell growth and proliferation to the initiation and regulation of immunological responses (50), many protein kinases were found to be decreased in TCMR in comparison with STA biopsies. For example, creatine kinase (CK), which is associated with reduced inflammation (51), decreased 7-fold in TCMR biopsies. The fourth group of proteins that significantly changed in their expression levels between the TCMR and STA biopsies is translation and transcription regulators (**Figure S2E**). Among them, HNRNPK, which could promote the activation of NLRP3 inflammasome (52), increased 2-fold in TCMR biopsies. The bioinformatic analysis demonstrated that the developed label-free-based proteomics method in this study not only could facilitate the understanding of the molecular mechanisms associated with TCMR, but also provide the potential biomarkers for disease diagnosis.

**Figure 6:**
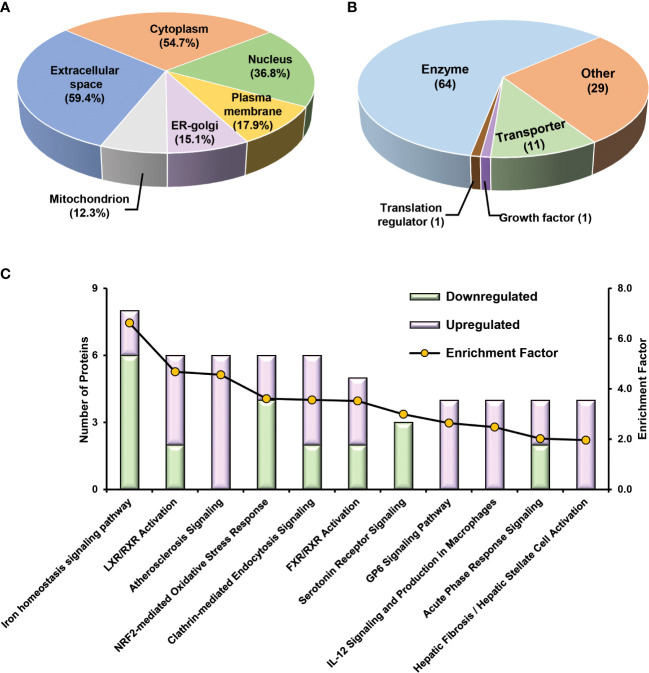
Bioinformatics analysis of 106 potential classifiers between TCMR and STA commonly quantified from two methods. Ingenuity Pathways Analysis of cDEPs commonly identified from both TMT- and label-free quantitative proteome analysis revealed cellular component **(A)**, molecular function **(B)**, and canonical pathways **(C)** enriched in the 106 potential classifiers between TCMR *vs.* STA samples. The orange and green labeled bars respectively represented the up-regulated and down-regulated proteins in TCMR in comparison with STA.

To obtain a panel of protein classifiers/biomarkers to diagnose TCMR with high accuracy, we chose the DEPs confidently quantified with the same trend (increase or decrease) in protein expression in both label-free-based- and TMT-based proteomics analyses. As a result, 106, 40, and 154 proteins were identified as potential classifiers for TCMR *vs*. STA, TCMR *vs*. BKPyVN, and BKPyVN *vs*. STA, respectively (**Supplementary Table S7**). The 106 potential classifiers/biomarkers between TCMR *vs*. STA were subjected to Ingenuity Pathway Analysis (IPA). The analysis revealed that the 106 potential classifiers/biomarkers were mainly located in extracellular exosome, nucleus, plasma membrane, ER-golgi and mitochondrion (**Figure 6A**), while more than 60% of them were enzymes (**Figure 6B**). In addition, these proteins were enriched in various signaling pathways associated with the inflammatory response, including iron homeostasis signaling pathway (53), energy production pathways (*e.g.*, galactose and sucrose degradation) (54), apoptosis pathways (*e.g.*, LXR/RXR and FXR/RXR activation) (55), and atherosclerosis signaling (56) (**Figure 6C** and **Supplementary Table S15**). In addition, these 106 potential classifiers/biomarkers between TCMR *vs*. STA are associated with kidney damage (e.g., ATP1B1, CST3, FAH, GSS, HPX and LYZ), tubule injury (e.g., CRYM, GSS, HAGH, HPX and LYZ) and kidney inflammation (e.g., DCN) (**Supplementary Table S15**). For example, CST3 (cystatin C), an extracellular space protein, was used as a biomarker to evaluate kidney function (glomerular filtration rate, GFR) (NCT00300066 in ClinicalTrials.gov database) and to predict the risk of ischemic stroke (NCT00479518). CST3 was also used as a potential biomarker to measure the efficacy of valsartan in the treatment of hypertension for patients with kidney dysfunction (NCT00140790). In addition to CST3, other proteins such as VIM (vimentin), LCP1 (lymphocyte cytosolic protein 1), and FTL (ferritin light chain) are also potential biomarkers for clinical diagnosis (**Table S9**). The above analysis showed that many proteins in the potential classifier panel were reported not only influences innate immunity but also determine T-cell-mediated immune response, demonstrating the feasibility of using this potential classifier panel in building TMCR disease prediction model.

We further evaluated whether a workflow integrating label-free quantitative proteomics technology with machine learning could be developed into a disease prediction tool for TCMR diagnosis. The protein intensity data (the summarized intensities of all identified peptides for each protein) in the FFPE biopsies of TCMR, BKPyVN and STA obtained from the label-free-based experiments was used as the classifiers for machine learning prediction model. As the core component of the developed prediction model, the selection of an optimal machine learning algorithm is prerequisite. LDA, Logistic Regression, Decision Tree, k-Nearest Neighbors, RF, SVM, Naive Bayes and Artificial Neural Network are among the commonly used machine learning techniques (57–59). In this study, three machine learning algorithms, LDA, SVM, and RF, were applied to the quantitative proteomics data collected from kidney FFPE biopsies. cDEPs identified in common from both TMT and label-free proteomic workflows were used as classifiers. With leave-one-out cross-validation, all three algorithms were found to achieve preliminary predictive performance for rejection with 100% sensitivity and specificity when applied to the discovery sample set. In addition, using an independent validation sample set of 5 TCMR and 5 STA biopsies, the TCMR prediction models also achieved satisfactory predictive power. A good prediction result was also achieved when the models were applied to transcriptome data published by others. Among the three models constructed in this study, RF-based TCMR prediction model outperformed the two other models. These results demonstrated the diagnostic potential of RF-based prediction model for kidney transplant injuries.

Although we applied stringent histopathologic criteria to define TCMR, rejection is a heterogenous process, and a larger sample size will be necessary to cover the broad spectrum of TCMR. Since no simple rule of thumb is available to determine the necessary sample size for omics studies seeking to find novel biomarkers, our study has limitations. The potential classifiers/biomarkers identified in this study will need to be optimized and validated using larger kidney rejection biopsy cohorts. The potential classifiers/biomarkers identified by mass spectrometry-based proteomic technology also need to be verified using other biomedical methods before being used to develop molecular tests. More accurate and specific molecular testing can lead to more effective treatment, prolong graft life, and improve the quality of life for patients with chronic kidney failure.

## Conclusion

Taken together, we have successfully developed an integrative pipeline by combining label-free quantitative proteomics and machine learning prediction models for TCMR diagnosis. Instead of relying on a single biomarker for disease diagnosis, we used a multi-biomarker panel to enhance diagnostic accuracy, sensitivity, and specificity. Because of the small sample size in this pilot study, the biomarker panel identified here will require further optimization and validation in larger biopsy data sets. As a proof-of-principle study, however, this report demonstrates the feasibility of clinical implementation of molecular diagnostics tests integrating label-free quantitative proteomics and machine learning predictive models.

## Data availability statement

The data presented in the study are deposited in the PRIDE repository, accession number PXD038601.

## Ethics statement

The studies involving human participants were reviewed and approved by University of Pittsburgh IRB (protocol 10110393). The ethics committee waived the requirement of written informed consent for participation.

## Author contributions

FF, PR, and KX designed the study. FF, PL, LS, PR, and KX conducted the study. FF, PL, LS, LM, XL, MR, GT, and KX performed data analyses. FF, PL, LS, PW, DB, PR, and KX interpreted the data. FF and KX wrote the manuscript with the support of PW, DB, LS, LM, XL, MR, and PR. All authors contributed to the article and approved the submitted version.

## References

[1] Collins AJ Foley RN Chavers B Gilbertson D Herzog C Ishani A. US Renal data system 2013 annual data report. Am J Kidney Dis (2014) 63(1):A7. doi: 10.1053/j.ajkd.2013.11.001 24360288

[2] Loupy A Aubert O Orandi BJ Naesens M Bouatou Y Raynaud M. Prediction system for risk of allograft loss in patients receiving kidney transplants: International derivation and validation study. BMJ (2019) 366:l4923. doi: 10.1136/bmj.l4923 31530561 PMC6746192

[3] Ghelichi-Ghojogh M Mohammadizadeh F Jafari F Vali M Jahanian S Mohammadi M. The global survival rate of graft and patient in kidney transplantation of children: A systematic review and meta-analysis. BMC Pediatr (2022) 22(1):503. doi: 10.1186/s12887-022-03545-2 36002803 PMC9404642

[4] Halloran PF. T Cell-mediated rejection of kidney transplants: A personal viewpoint. Am J Transplant (2010) 10(5):1126–34. doi: 10.1111/j.1600-6143.2010.03053.x 20346061

[5] Roufosse C Simmonds N Clahsen-van Groningen M Haas M Henriksen KJ Horsfield C. A 2018 reference guide to the banff classification of renal allograft pathology. Transplantation (2018) 102(11):1795–814. doi: 10.1097/TP.0000000000002366 PMC7597974 30028786

[6] Bobka S Ebert N Koertvely E Jacobi J Wiesener M Buttner-Herold M. Is early complement activation in renal transplantation associated with later graft outcome? Kidney Blood Press Res (2018) 43(5):1488–504. doi: 10.1159/000494014 30286468

[7] Randhawa P. The molecular microscope (MMDX(R)) interpretation of thoracic and abdominal allograft biopsies: Putting things in perspective for the clinician. Clin Transpl (2021) 35(4):e14223. doi: 10.1111/ctr.14223 33755254

[8] Randhawa P. The MMDx((R)) diagnostic system: A critical re-appraisal of its knowledge gaps and a call for rigorous validation studies. Clin Transplant (2022) 36(11):e14747. doi: 10.1111/ctr.14747 35678044

[9] Pan L Lyu Z Adam B Zeng G Wang Z Huang Y. Polyomavirus BK nephropathy-associated transcriptomic signatures: A critical reevaluation. Transplant Direct (2018) 4(2):e339. doi: 10.1097/TXD.0000000000000752 29464200 PMC5811268

[10] Drachenberg CB Papadimitriou JC Hirsch HH Wali R Crowder C Nogueira J. Histological patterns of polyomavirus nephropathy: Correlation with graft outcome and viral load. Am J Transpl (2004) 4(12):2082–92. doi: 10.1046/j.1600-6143.2004.00603.x 15575913

[11] Wynants L Van Calster B Collins GS Riley RD Heinze G Schuit E. Prediction models for diagnosis and prognosis of covid-19: Systematic review and critical appraisal. BMJ (2020) 369:m1328. doi: 10.1136/bmj.m1328 32265220 PMC7222643

[12] Park DJ Park MW Lee H Kim YJ Kim Y Park YH. Development of machine learning model for diagnostic disease prediction based on laboratory tests. Sci Rep (2021) 11(1):7567. doi: 10.1038/s41598-021-87171-5 33828178 PMC8026627

[13] Cruz JA Wishart DS. Applications of machine learning in cancer prediction and prognosis. Cancer Inform (2007) 2:59–77. 19458758 PMC2675494

[14] Liu P Tseng G Wang Z Huang Y Randhawa P. Diagnosis of T-cell-mediated kidney rejection in formalin-fixed, paraffin-embedded tissues using RNA-seq-based machine learning algorithms. Hum Pathol (2019) 84:283–90. doi: 10.1016/j.humpath.2018.09.013 30296518

[15] Song L Fang F Liu P Zeng G Liu H Zhao Y. Quantitative proteomics for monitoring renal transplant injury. Proteomics Clin Appl (2020) 14(4):e1900036. doi: 10.1002/prca.201900036 31999393

[16] Zeng G Huang Y Huang Y Lyu Z Lesniak D Randhawa P. Antigen-specificity of T cell infiltrates in biopsies with T cell-mediated rejection and BK polyomavirus viremia: Analysis by next generation sequencing. Am J Transpl (2016) 16(11):3131–8. doi: 10.1111/ajt.13911 PMC5083170 27273900

[17] Loupy A Haas M Roufosse C Naesens M Adam B Afrouzian M. The banff 2019 kidney meeting report (I): Updates on and clarification of criteria for T cell- and antibody-mediated rejection. Am J Transpl (2020) 20(9):2318–31. doi: 10.1111/ajt.15898 PMC7496245 32463180

[18] Xiao K Zhao Y Choi M Liu H Blanc A Qian J. Revealing the architecture of protein complexes by an orthogonal approach combining HDXMS, CXMS, and disulfide trapping. Nat Protoc (2018) 13(6):1403–28. doi: 10.1038/nprot.2018.037 29844522

[19] Zhao Y Xiao K. Proteomic analysis of the beta-arrestin interactomes. Methods Mol Biol (2019) 1957:217–32. doi: 10.1007/978-1-4939-9158-7_14 30919357

[20] Rappsilber J Ishihama Y Mann M. Stop and go extraction tips for matrix-assisted laser desorption/ionization, nanoelectrospray, and LC/MS sample pretreatment in proteomics. Anal Chem (2003) 75(3):663–70. doi: 10.1021/ac026117i 12585499

[21] Wisniewski JR Zougman A Nagaraj N Mann M. Universal sample preparation method for proteome analysis. Nat Methods (2009) 6(5):359–62. doi: 10.1038/nmeth.1322 19377485

[22] Chawade A Alexandersson E Levander F. Normalyzer: a tool for rapid evaluation of normalization methods for omics data sets. J Proteome Res (2014) 13(6):3114–20. doi: 10.1021/pr401264n PMC4053077 24766612

[23] Tyanova S Temu T Sinitcyn P Carlson A Hein MY Geiger T. The Perseus computational platform for comprehensive analysis of (prote)omics data. Nat Methods (2016) 13(9):731–40. doi: 10.1038/nmeth.3901 27348712

[24] Alter O Brown PO Botstein D. Singular value decomposition for genome-wide expression data processing and modeling. Proc Natl Acad Sci USA (2000) 97(18):10101–6. doi: 10.1073/pnas.97.18.10101 PMC27718 10963673

[25] Ritchie ME Phipson B Wu D Hu Y Law CW Shi W. Limma powers differential expression analyses for RNA-sequencing and microarray studies. Nucleic Acids Res (2015) 43(7):e47. doi: 10.1093/nar/gkv007 25605792 PMC4402510

[26] Pineda S Sigdel TK Chen J Jackson AM Sirota M Sarwal MM. Corrigendum: Novel non-histocompatibility antigen mismatched variants improve the ability to predict antibody-mediated rejection risk in kidney transplant. Front Immunol (2018) 9:107. doi: 10.3389/fimmu.2018.00107 29406538 PMC5797733

[27] Huang da W Sherman BT Lempicki RA. Systematic and integrative analysis of large gene lists using DAVID bioinformatics resources. Nat Protoc (2009) 4(1):44–57. doi: 10.1038/nprot.2008.211 19131956

[28] Huang da W Sherman BT Lempicki RA. Bioinformatics enrichment tools: paths toward the comprehensive functional analysis of large gene lists. Nucleic Acids Res (2009) 37(1):1–13. doi: 10.1093/nar/gkn923 19033363 PMC2615629

[29] Shashoa NAA Salem NA Jleta IN Abusaeeda O. Classification depend on linear discriminant analysis using desired outputs, in: Proceedings in the 2016 17th International Conference on Sciences and Techniques of Automatic Control and Computer Engineering (STA), (Sousse, Tunisia: New York: IEEE) (2016). 19–21 p.

[30] Tang YC. Deep learning using linear support vector machines. Challenges in Representation Learning Workshop (Atlanta, GA, USA: ICML) (2013).

[31] Saffari A Leistner C Santner J Godec M Bischof H. On-line random forests, in: 2009 IEEE 12th International Conference on Computer Vision Workshops, ICCV Workshops, (Koyoto, Japan: IEEE) (2009). 1393–1400 p.

[32] Shao Z Er MJ. Efficient leave-One-Out cross-validation-based regularized extreme learning machine. Neurocomputing (2016) 194:260–70. doi: 10.1016/j.neucom.2016.02.058 26259254

[33] Cawley GC Talbot NL. Fast exact leave-one-out cross-validation of sparse least-squares support vector machines. Neural Netw (2004) 17(10):1467–75. doi: 10.1016/j.neunet.2004.07.002 15541948

[34] Kearns M Ron D. Algorithmic stability and sanity-check bounds for leave-one-out cross-validation. Neural Comput (1999) 11(6):1427–53. doi: 10.1162/089976699300016304 10423502

[35] Wu J Mei J Wen S Liao S Chen J Shen Y. A self-adaptive genetic algorithm-artificial neural network algorithm with leave-one-out cross validation for descriptor selection in QSAR study. J Comput Chem (2010) 31(10):1956–68. doi: 10.1002/jcc.21471 20512843

[36] Hajian-Tilaki K. Receiver operating characteristic (ROC) curve analysis for medical diagnostic test evaluation. Caspian J Intern Med (2013) 4(2):627–35. PMC3755824 24009950

[37] Hanley JA McNeil BJ. The meaning and use of the area under a receiver operating characteristic (ROC) curve. Radiology (1982) 143(1):29–36. doi: 10.1148/radiology.143.1.7063747 7063747

[38] Halloran PF Pereira AB Chang J Matas A Picton M De Freitas D. Potential impact of microarray diagnosis of T cell-mediated rejection in kidney transplants: The INTERCOM study. Am J Transpl (2013) 13(9):2352–63. doi: 10.1111/ajt.12387 23915426

[39] Reeve J Sellares J Mengel M Sis B Skene A Hidalgo L. Molecular diagnosis of T cell-mediated rejection in human kidney transplant biopsies. Am J Transpl (2013) 13(3):645–55. doi: 10.1111/ajt.12079 23356949

[40] Zhang P Lehmann BD Shyr Y Guo Y. The utilization of formalin fixed-Paraffin-Embedded specimens in high throughput genomic studies. Int J Genomics (2017) 2017:1926304. doi: 10.1155/2017/1926304 28246590 PMC5299160

[41] Vonbrunn E Ries T Sollner S Muller-Deile J Buttner-Herold M Amann K. Multiplex gene analysis reveals T-cell and antibody-mediated rejection-specific upregulation of complement in renal transplants. Sci Rep (2021) 11(1):15464. doi: 10.1038/s41598-021-94954-3 34326417 PMC8322413

[42] Piehowski PD Petyuk VA Sontag RL Gritsenko MA Weitz KK Fillmore TL. Residual tissue repositories as a resource for population-based cancer proteomic studies. Clin Proteomics (2018) 15:26. doi: 10.1186/s12014-018-9202-4 30087585 PMC6074037

[43] Decramer S Gonzalez de Peredo A Breuil B Mischak H Monsarrat B Bascands JL. Urine in clinical proteomics. Mol Cell Proteomics (2008) 7(10):1850–62. doi: 10.1074/mcp.R800001-MCP200 18667409

[44] Rai AJ Gelfand CA Haywood BC Warunek DJ Yi J Schuchard MD. HUPO plasma proteome project specimen collection and handling: towards the standardization of parameters for plasma proteome samples. Proteomics (2005) 5(13):3262–77. doi: 10.1002/pmic.200401245 16052621

[45] Anderson NL Anderson NG. The human plasma proteome: history, character, and diagnostic prospects. Mol Cell Proteomics (2002) 1(11):845–67. doi: 10.1074/mcp.R200007-MCP200 12488461

[46] Liebermeister W Noor E Flamholz A Davidi D Bernhardt J Milo R. Visual account of protein investment in cellular functions. Proc Natl Acad Sci USA (2014) 111(23):8488–93. doi: 10.1073/pnas.1314810111 PMC4060655 24889604

[47] Franco B Mueller HY Li C Snopkowski C Dadhania DM Xiang JZ. Adaptive immune landscape of T-cell mediated rejection of human kidney allografts. bioRxiv (2022). doi: 10.1101/2022.05.15.492021

[48] Eisenhut M. Changes in ion transport in inflammatory disease. J Inflamm (Lond) (2006) 3:5. doi: 10.1186/1476-9255-3-5 16571116 PMC1562419

[49] Domingo-Fernandez R Coll RC Kearney J Breit S O'Neill LAJ. The intracellular chloride channel proteins CLIC1 and CLIC4 induce IL-1beta transcription and activate the NLRP3 inflammasome. J Biol Chem (2017) 292(29):12077–87. doi: 10.1074/jbc.M117.797126 PMC5519359 28576828

[50] Karin M. Inflammation-activated protein kinases as targets for drug development. Proc Am Thorac Soc (2005) 2(4):386–90; discussion 94-5. doi: 10.1513/pats.200504-034SR 16267367 PMC2713329

[51] Bekkelund SI Johnsen SH. Creatine kinase is associated with reduced inflammation in a general population: The tromso study. PloS One (2018) 13(5):e0198133. doi: 10.1371/journal.pone.0198133 29813131 PMC5973606

[52] Feng J Li H Li J Meng P Wang L Liu C. hnRNPK knockdown alleviates NLRP3 inflammasome priming by repressing FLIP expression in Raw264.7 Macrophages. Redox Rep (2020) 25(1):104–11. doi: 10.1080/13510002.2020.1857157 PMC7717877 33269646

[53] Bonaccorsi-Riani E Danger R Lozano JJ Martinez-Picola M Kodela E Mas-Malavila R. Iron deficiency impairs intra-hepatic lymphocyte mediated immune response. PloS One (2015) 10(8):e0136106. doi: 10.1371/journal.pone.0136106 26287688 PMC4542211

[54] Delmastro-Greenwood MM Piganelli JD. Changing the energy of an immune response. Am J Clin Exp Immunol (2013) 2(1):30–54. 23885324 PMC3714201

[55] Ekert PG Vaux DL. Apoptosis and the immune system. Br Med Bull (1997) 53(3):591–603. doi: 10.1093/oxfordjournals.bmb.a011632 9374039

[56] Lei ZN Teng QX Tian Q Chen W Xie Y Wu K. Signaling pathways and therapeutic interventions in gastric cancer. Signal Transduct Target Ther (2022) 7(1):358. doi: 10.1038/s41392-022-01190-w 36209270 PMC9547882

[57] Hassan M Butt A Baba M. Logistic regression versus neural networks: The best accuracy in prediction of diabetes disease. (Coimbatore, India: The Research Publication) (2017). 33–42 p.

[58] Khanna D Sahu R Baths V Deshpande B. Comparative study of classification techniques (SVM, logistic regression and neural networks) to predict the prevalence of heart disease. Int J Mach Learn Computing (2015) 5:414–9. doi: 10.7763/IJMLC.2015.V5.544

[59] Muthuvel M Sivaraju D Ramamoorthy G. Analysis of heart disease prediction using various machine learning techniques. (Coimbatore, India: Springer Cham) (2019).

