# Supplementary material for: Diagnosis of T-cell-mediated kidney rejection by biopsy-based proteomic biomarkers and machine learning
Source: Front Immunol. 2023 Feb 6;14:1090373. doi: 10.3389/fimmu.2023.1090373 (PMC9939643; doi:10.3389/fimmu.2023.1090373)
Supplement: Supplementary file 1 [file DataSheet_1.docx]

**Supplemental Material**

1. **Materials and Methods**

**Machine learning and validation**

There are two microarray-based transcriptome data sets, GSE48581 [1] and GSE36059 ([2], applied for the evaluation of the developed predictive model. A total of 306 samples were used in GSE48581, among which 254 (222 STA and 32 TCMR) samples were used in our validation experiment. In GSE36059, 316 (281 STA and 35 TCMR) out of 411 samples were used in our validation experiment.

1. **Supplementary Figures**

**Figure S1. Diagnostic ability of the three different predictive models applied to distinguish TCMR from other phenotypes.** Receiver operator curve (ROC) analysis for kidney allograft injuries panel provided AUCs of 1, meaning that 100% specificity and 100% sensitivity were achieved with the developed model for distinguishing disease phenotypes.

**Figure S2. The bioinformatics analysis of the DEPs obtained by label-free proteomics data for TCMR.** **(A)** The 329 DEPs between TCMR and STA obtained by label-free proteomics data were subjected to STRING software (https://string-db.org/), with the protein interaction and function distribution obtained. **(B-E)** The functional enrichment information of the 329 DEPs between TCMR and STA quantified from label-free proteomics data were obtained via DAVID analysis, with the inflammation-associated proteins extracted. On the y axis, a fold change value of 0 corresponds to no difference in expression levels between TCMR and STA biopsies. For values plotted above 0, protein abundance in TCMR is higher than in STA. For values plotted below 0, protein abundance in STA is higher than in TCMR. Fold change was calculated using edgeR. The p-value is the probability value adjusted for FDR using the Benjamini-Hochberg method. The x axis is the log10 p-values; for example, a value of -5 corresponds to p-value = 0.00001. Proteins expression patterns for those involved in **(B)** collagens, **(C)** ion channel and transporter proteins, **(D)** kinases and **(E)** translation and transcription regulators.


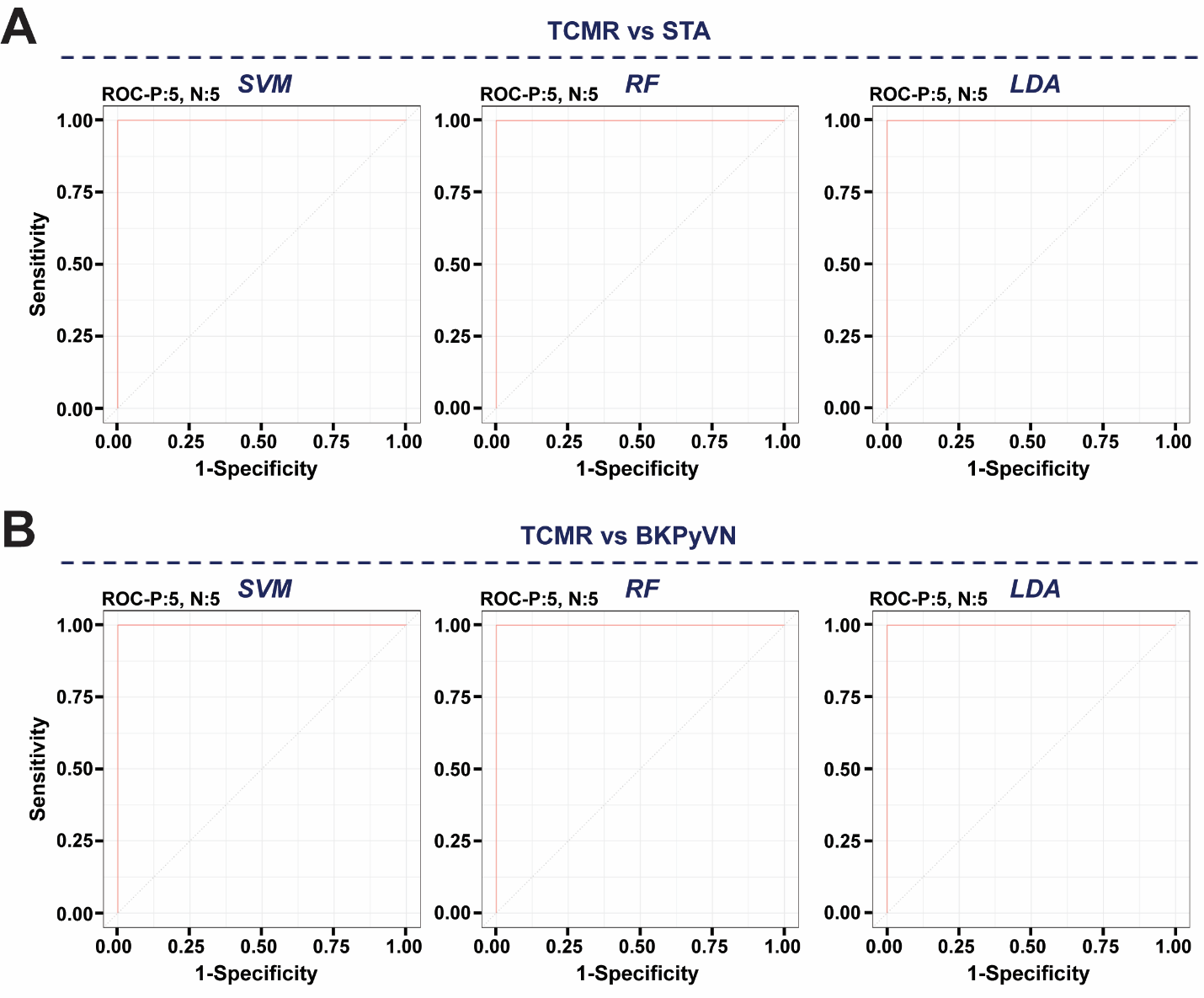


**Figure S1**

**
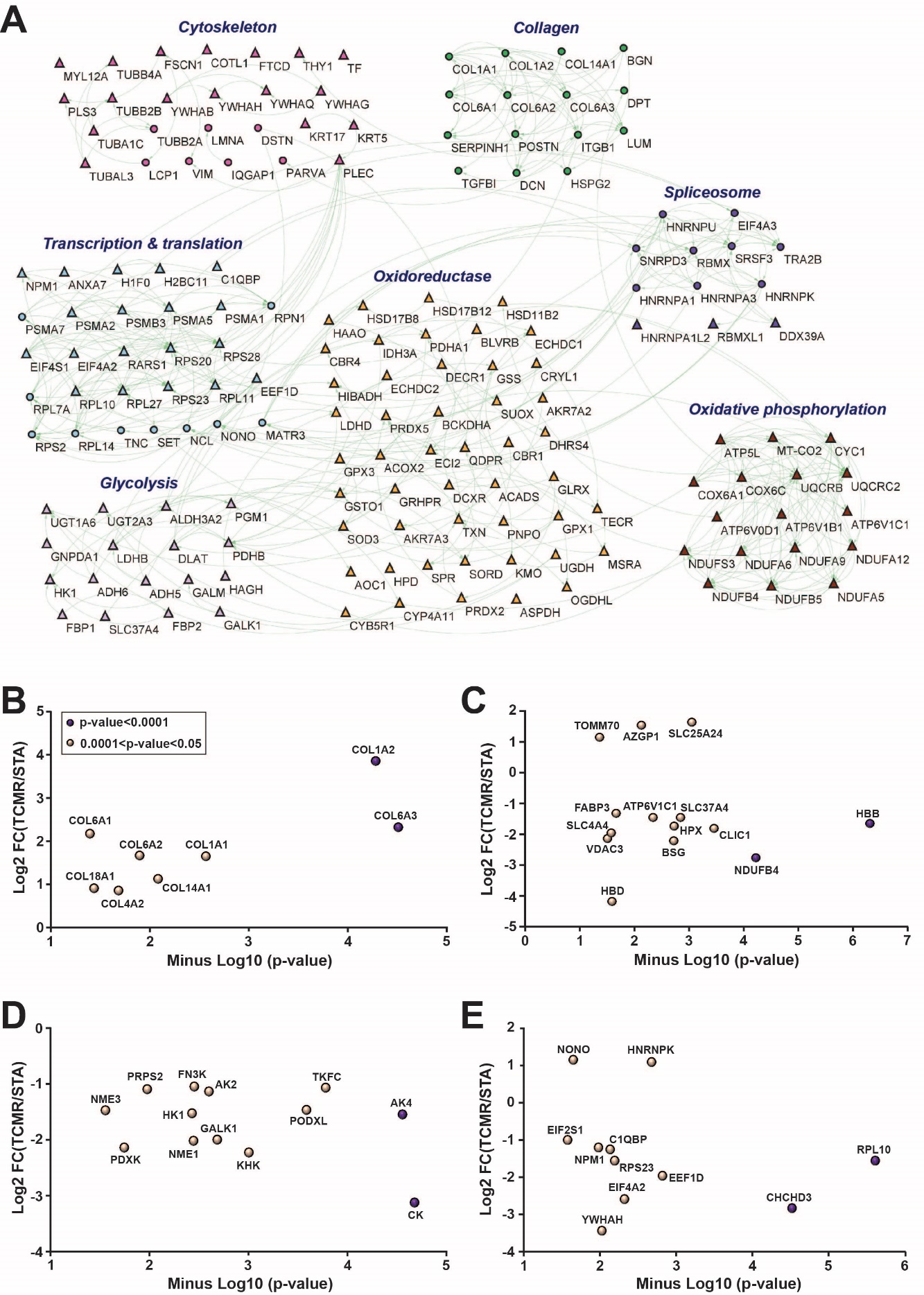
Figure S2**

**References**

**Note: This list also includes references for Table S8 and S9.**

1. Halloran, P.F., et al., *Potential impact of microarray diagnosis of T cell-mediated rejection in kidney transplants: The INTERCOM study.* Am J Transplant, 2013. **13**(9): p. 2352-63.

2. Reeve, J., et al., *Molecular diagnosis of T cell-mediated rejection in human kidney transplant biopsies.* Am J Transplant, 2013. **13**(3): p. 645-55.

3. Famulski, K.S., et al., *Kidney transplants with progressing chronic diseases express high levels of acute kidney injury transcripts.* Am J Transplant, 2013. **13**(3): p. 634-44.

4. Wang, X., et al., *Physiological functions of ferroportin in the regulation of renal iron recycling and ischemic acute kidney injury.* Am J Physiol Renal Physiol, 2018. **315**(4): p. F1042-F1057.

5. Kheir, V., et al., *Mutation update: TGFBI pathogenic and likely pathogenic variants in corneal dystrophies.* Hum Mutat, 2019. **40**(6): p. 675-693.

6. Horpacsy, G., et al., *Changes in serum and urine lysozyme activity after kidney transplantation: influence of graft function and therapy with azathioprine.* Clin Chem, 1978. **24**(1): p. 74-9.

7. Ayub, S., et al., *Evaluation of renal function by cystatin C in renal transplant recipients.* Exp Clin Transplant, 2014. **12**(1): p. 37-40.

8. Stubbe, J., et al., *Identification of differential gene expression patterns in human arteries from patients with chronic kidney disease.* Am J Physiol Renal Physiol, 2018. **314**(6): p. F1117-F1128.

9. Hruba, P., et al., *Molecular Fingerprints of Borderline Changes in Kidney Allografts Are Influenced by Donor Category.* Front Immunol, 2020. **11**: p. 423.

10. Wan, F., et al., *Upregulation of COL6A1 is predictive of poor prognosis in clear cell renal cell carcinoma patients.* (1949-2553 (Electronic)).

11. Halloran, P.F., et al., *Review: The transcripts associated with organ allograft rejection.* Am J Transplant, 2018. **18**(4): p. 785-795.

12. Liu, J., et al., *Molecular characterization of the transition from acute to chronic kidney injury following ischemia/reperfusion.* JCI Insight, 2017. **2**(18).

13. Satirapoj, B., et al., *Urine periostin as a biomarker of renal injury in chronic allograft nephropathy.* Transplant Proc, 2014. **46**(1): p. 135-40.

14. Schaefer, L., *Small leucine-rich proteoglycans in kidney disease.* J Am Soc Nephrol, 2011. **22**(7): p. 1200-7.

15. van Swelm, R.P.L., J.F.M. Wetzels, and D.W. Swinkels, *The multifaceted role of iron in renal health and disease.* Nat Rev Nephrol, 2020. **16**(2): p. 77-98.

16. Gonzalez-Salvatierra, S., et al., *Osteoglycin as a Potential Biomarker of Mild Kidney Function Impairment in Type 2 Diabetes Patients.* J Clin Med, 2021. **10**(10).

17. Domingos, M.A., et al., *Urinary Retinol-Binding Protein: Relationship to Renal Function and Cardiovascular Risk Factors in Chronic Kidney Disease.* PLoS One, 2016. **11**(9): p. e0162782.

18. Bi, H., et al., *The mRNA landscape profiling reveals potential biomarkers associated with acute kidney injury AKI after kidney transplantation.* PeerJ, 2020. **8**: p. e10441.

19. Ozdemir, B.H., et al., *Relationships between HLA-A, -B, -DQ and -DR antigens and interstitial fibrosis in renal allografts.* Ren Fail, 2004. **26**(3): p. 243-6.

20. Fu, H., et al., *Tenascin-C Is a Major Component of the Fibrogenic Niche in Kidney Fibrosis.* J Am Soc Nephrol, 2017. **28**(3): p. 785-801.

21. Cheng, C.W., et al., *Serum ApoA4 levels predicted the progression of renal impairment in T2DM.* Eur J Clin Invest, 2018. **48**(6): p. e12937.

22. Neymeyer, H., et al., *Activation of annexin A1 signalling in renal fibroblasts exerts antifibrotic effects.* Acta Physiol (Oxf), 2015. **215**(3): p. 144-58.

23. Pongsakul, N., et al., *Lamin A/C in renal tubular cells is important for tissue repair, cell proliferation, and calcium oxalate crystal adhesion, and is associated with potential crystal receptors.* FASEB J, 2016. **30**(10): p. 3368-3377.

24. Besarani, D., et al., *Role of anti-vimentin antibodies in renal transplantation.* (1534-6080 (Electronic)).

25. Wang, M., *ApoC3 fires up monocytes to promote tissue damage.* Nat Rev Nephrol, 2020. **16**(3): p. 131.

26. Xu, J., et al., *Identification of blood-based key biomarker and immune infiltration in Immunoglobulin A nephropathy by comprehensive bioinformatics analysis and a cohort validation.* J Transl Med, 2022. **20**(1): p. 145.

27. Osis, G., et al., *Expression of lactate dehydrogenase A and B isoforms in the mouse kidney.* Am J Physiol Renal Physiol, 2021. **320**(5): p. F706-F718.

28. Liu, D., et al., *Identification of Key Genes and Candidated Pathways in Human Autosomal Dominant Polycystic Kidney Disease by Bioinformatics Analysis.* Kidney Blood Press Res, 2019. **44**(4): p. 533-552.

29. Kuo, C.W., et al., *Serum and urinary SOD3 in patients with type 2 diabetes: comparison with early chronic kidney disease patients and association with development of diabetic nephropathy.* Am J Physiol Renal Physiol, 2019. **316**(1): p. F32-F41.

30. Audrito, V., V.G. Messana, and S. Deaglio, *NAMPT and NAPRT: Two Metabolic Enzymes With Key Roles in Inflammation.* Front Oncol, 2020. **10**: p. 358.

31. Schwarz, C., et al., *Complete renal tubular acidosis late after kidney transplantation.* Nephrol Dial Transplant, 2006. **21**(9): p. 2615-20.

32. van Ginkel, W.G., et al., *Long-Term Outcomes and Practical Considerations in the Pharmacological Management of Tyrosinemia Type 1.* Paediatr Drugs, 2019. **21**(6): p. 413-426.

33. Perco, P., et al., *Identification of dicarbonyl and L-xylulose reductase as a therapeutic target in human chronic kidney disease.* JCI Insight, 2019. **4**(12).

34. Dhondup, T., et al., *Combined Liver-Kidney Transplantation for Primary Hyperoxaluria Type 2: A Case Report.* Am J Transplant, 2018. **18**(1): p. 253-257.

35. Ketz, J., et al., *Developmental loss, but not pharmacological suppression, of renal carbonic anhydrase 2 results in pyelonephritis susceptibility.* Am J Physiol Renal Physiol, 2020. **318**(6): p. F1441-F1453.

36. Tinel, C., et al., *Integrative Omics Analysis Unravels Microvascular Inflammation-Related Pathways in Kidney Allograft Biopsies.* Front Immunol, 2021. **12**: p. 738795.

37. Grunebaum, E., et al., *Partial Purine Nucleoside Phosphorylase Deficiency Helps Determine Minimal Activity Required for Immune and Neurological Development.* Front Immunol, 2020. **11**: p. 1257.

38. Hashimoto, S., et al., *Lysophosphatidic acid activates Arf6 to promote the mesenchymal malignancy of renal cancer.* Nat Commun, 2016. **7**: p. 10656.

39. Sigdel, T.K., et al., *Shotgun proteomics identifies proteins specific for acute renal transplant rejection.* Proteomics Clin Appl, 2010. **4**(1): p. 32-47.

40. Zacchia, M., et al., *Proteomics and metabolomics studies exploring the pathophysiology of renal dysfunction in autosomal dominant polycystic kidney disease and other ciliopathies.* Nephrol Dial Transplant, 2019.

41. Zhai, X., H. Lou, and J. Hu, *Five-gene signature predicts acute kidney injury in early kidney transplant patients.* Aging (Albany NY), 2022. **14**(6): p. 2628-2644.

42. Radon, V., et al., *Ubiquitin C-Terminal Hydrolase L1 is required for regulated protein degradation through the ubiquitin proteasome system in kidney.* Kidney Int, 2018. **93**(1): p. 110-127.

43. Joist, H., D.C. Brennan, and D.W. Coyne, *Anemia in the kidney-transplant patient.* Adv Chronic Kidney Dis, 2006. **13**(1): p. 4-10.

44. Hartmannova, H., et al., *Acadian variant of Fanconi syndrome is caused by mitochondrial respiratory chain complex I deficiency due to a non-coding mutation in complex I assembly factor NDUFAF6.* Hum Mol Genet, 2016. **25**(18): p. 4062-4079.

45. Larson, E.L., et al., *Fumarylacetoacetate hydrolase gene as a knockout target for hepatic chimerism and donor liver production.* Stem Cell Reports, 2021. **16**(11): p. 2577-2588.

46. Krikken, J.A., et al., *High plasma hemopexin activity is an independent risk factor for late graft failure in renal transplant recipients.* Transpl Int, 2010. **23**(8): p. 805-12.

47. Zhu, Y., et al., *Hepatic GALE Regulates Whole-Body Glucose Homeostasis by Modulating Tff3 Expression.* (1939-327X (Electronic)).

48. Pacal, L., et al., *Deleterious Effect of Advanced CKD on Glyoxalase System Activity not Limited to Diabetes Aetiology.* Int J Mol Sci, 2018. **19**(5).

49. Wong, T.Y., et al., *Glucose-mediated induction of TGF-beta 1 and MCP-1 in mesothelial cells in vitro is osmolality and polyol pathway dependent.* Kidney Int, 2003. **63**(4): p. 1404-16.

50. Akhtar, M.Z., et al., *Using an Integrated -Omics Approach to Identify Key Cellular Processes That Are Disturbed in the Kidney After Brain Death.* Am J Transplant, 2016. **16**(5): p. 1421-40.

51. Arndt, T., et al., *Increased serum concentration of carbohydrate-deficient transferrin in patients with combined pancreas and kidney transplantation.* Clin Chem, 1997. **43**(2): p. 344-51.

52. Bignon, Y., et al., *Cell stress response impairs de novo NAD+ biosynthesis in the kidney.* JCI Insight, 2022. **7**(1).

53. Lacour, B., et al., *Pyridoxal 5′-phosphate deficiency in uremic undialyzed, hemodialyzed, and non-uremic kidney transplant patients.* Clinica Chimica Acta, 1983. **127**(2): p. 205-215.

54. Lassiter, R., et al., *Protective Role of Kynurenine 3-Monooxygenase in Allograft Rejection and Tubular Injury in Kidney Transplantation.* Front Immunol, 2021. **12**: p. 671025.

55. Newnham, T., et al., *Liver transplantation for argininosuccinic aciduria: clinical, biochemical, and metabolic outcome.* Liver Transpl, 2008. **14**(1): p. 41-5.

56. Kwon, J.H., et al., *Upregulation of Carbonyl Reductase 1 by Nrf2 as a Potential Therapeutic Intervention for Ischemia/ Reperfusion Injury during Liver Transplantation.* Mol Cells, 2019. **42**(9): p. 672-685.

57. Miura, K., et al., *Mutational analyses of the ATP6V1B1 and ATP6V0A4 genes in patients with primary distal renal tubular acidosis.* Nephrol Dial Transplant, 2013. **28**(8): p. 2123-30.

58. Kelly, T.N., et al., *The role of renin-angiotensin-aldosterone system genes in the progression of chronic kidney disease: findings from the Chronic Renal Insufficiency Cohort (CRIC) study.* Nephrol Dial Transplant, 2015. **30**(10): p. 1711-8.

59. Harper, J.D., et al., *Incidence of increased creatine kinase and its effect on kidney function in hand assisted laparoscopic kidney donors and their recipients.* J Urol, 2007. **178**(4 Pt 1): p. 1391-5; discussion 1395.

60. Ibai Los-Arcos*1, L.M., Francesc Canals2, Francesc Moreso3, Lluis Girado4, Marta Crespo5, Nuria Sabe6, Oriol Bestard7, Gema Ariceta8, Manel Perello3, Joan Gavaldà I Santapau9, Oscar Len1, *Determination of BK virus nephropathy biomarkers in urine samples from kidney transplant recipients by proteomics* 27th European Congress of Clinical Microbiology and Infectious Diseases (ECCMID), 2017. **2017**.

61. Ren, F., et al., *Quantitative proteomics identification of phosphoglycerate mutase 1 as a novel therapeutic target in hepatocellular carcinoma.* Mol Cancer, 2010. **9**: p. 81.

62. Aicher, L., et al., *New insights into cyclosporine A nephrotoxicity by proteome analysis.* (0173-0835 (Print)).

63. Yuan, L., et al., *Carbonic Anhydrase 1-Mediated Calcification Is Associated With Atherosclerosis, and Methazolamide Alleviates Its Pathogenesis.* Front Pharmacol, 2019. **10**: p. 766.

64. Vallabhajosyula, P., et al., *Tissue-specific exosome biomarkers for noninvasively monitoring immunologic rejection of transplanted tissue.* J Clin Invest, 2017. **127**(4): p. 1375-1391.

65. Reichold, M., et al., *Glycine Amidinotransferase (GATM), Renal Fanconi Syndrome, and Kidney Failure.* J Am Soc Nephrol, 2018. **29**(7): p. 1849-1858.

66. Watanabe, H., et al., *Expression of Acsm2, a kidney-specific gene, parallels the function and maturation of proximal tubular cells.* Am J Physiol Renal Physiol, 2020. **319**(4): p. F603-F611.

67. Quesada, A., et al., *Urinary aminopeptidase activities as early and predictive biomarkers of renal dysfunction in cisplatin-treated rats.* PLoS One, 2012. **7**(7): p. e40402.

68. Yepes-Calderon, M., et al., *Urinary liver-type fatty acid-binding protein is independently associated with graft failure in outpatient kidney transplant recipients.* Am J Transplant, 2021. **21**(4): p. 1535-1544.

69. Hemmingsen, C., et al., *Regulation of renal calbindin-D28K: the role of calcitonin.* Calcif Tissue Int, 1995. **56**(5): p. 372-5.

70. Wijermars, L.G.M., et al., *Association of Impaired Reactive Aldehyde Metabolism with Delayed Graft Function in Human Kidney Transplantation.* Oxid Med Cell Longev, 2018. **2018**: p. 3704129.

71. Stanfill, A., et al., *A Pilot Study of Demographic and Dopaminergic Genetic Contributions to Weight Change in Kidney Transplant Recipients.* PLoS One, 2015. **10**(9): p. e0138885.

72. Gervasini, G., et al., *A 3'-UTR Polymorphism in Soluble Epoxide Hydrolase Gene Is Associated with Acute Rejection in Renal Transplant Recipients.* PLoS One, 2015. **10**(7): p. e0133563.

73. Olyaei, A., et al., *The efficacy and safety of the 3-hydroxy-3-methylglutaryl-CoA reductase inhibitors in chronic kidney disease, dialysis, and transplant patients.* Clin J Am Soc Nephrol, 2011. **6**(3): p. 664-78.

74. From the American Association of Neurological Surgeons, A.S.o.N.C., et al., *Multisociety Consensus Quality Improvement Revised Consensus Statement for Endovascular Therapy of Acute Ischemic Stroke.* Int J Stroke, 2018. **13**(6): p. 612-632.

75. Gupta, I.R., et al., *ARHGDIA: a novel gene implicated in nephrotic syndrome.* J Med Genet, 2013. **50**(5): p. 330-8.

76. Bloomer, J., et al., *Molecular defects in ferrochelatase in patients with protoporphyria requiring liver transplantation.* J Clin Invest, 1998. **102**(1): p. 107-14.

77. Ferraresso, M., et al., *Relationship between mRNA expression levels of CYP3A4, CYP3A5 and SXR in peripheral mononuclear blood cells and aging in young kidney transplant recipients under tacrolimus treatment.* Pharmacogenomics, 2015. **16**(5): p. 483-91.

78. Shin, H., et al., *Longitudinal analysis of whole blood transcriptomes to explore molecular signatures associated with acute renal allograft rejection.* Bioinform Biol Insights, 2014. **8**: p. 17-33.

79. Lee, P.S., et al., *Plasma gelsolin and circulating actin correlate with hemodialysis mortality.* J Am Soc Nephrol, 2009. **20**(5): p. 1140-8.

80. Kurian, S.M., et al., *Biomarkers for early and late stage chronic allograft nephropathy by proteogenomic profiling of peripheral blood.* PLoS One, 2009. **4**(7): p. e6212.

81. Melis, N., et al., *Targeting eIF5A Hypusination Prevents Anoxic Cell Death through Mitochondrial Silencing and Improves Kidney Transplant Outcome.* J Am Soc Nephrol, 2017. **28**(3): p. 811-822.

82. Bronze-da-Rocha, E. and A. Santos-Silva, *Neutrophil Elastase Inhibitors and Chronic Kidney Disease.* Int J Biol Sci, 2018. **14**(10): p. 1343-1360.

83. Wang, J., et al., *The correlation between the expression of genes involved in drug metabolism and the blood level of tacrolimus in liver transplant receipts.* Sci Rep, 2017. **7**(1): p. 3429.

84. Zhou, X., et al., *Ribosomal proteins: functions beyond the ribosome.* J Mol Cell Biol, 2015. **7**(2): p. 92-104.

85. Petrova, D.T., et al., *Effects of mycophenolate mofetil on kidney function and phosphorylation status of renal proteins in Alport COL4A3-deficient mice.* Proteome Sci, 2014. **12**(1): p. 56.

86. Lin, T.C., *DDX3X Multifunctionally Modulates Tumor Progression and Serves as a Prognostic Indicator to Predict Cancer Outcomes.* Int J Mol Sci, 2019. **21**(1).

87. Zhou, J., et al., *Bortezomib attenuates renal interstitial fibrosis in kidney transplantation via regulating the EMT induced by TNF-alpha-Smurf1-Akt-mTOR-P70S6K pathway.* J Cell Mol Med, 2019. **23**(8): p. 5390-5402.

88. Soderholm, J.F., et al., *Importazole, a small molecule inhibitor of the transport receptor importin-beta.* ACS Chem Biol, 2011. **6**(7): p. 700-8.

89. Lozano, J.J., et al., *Comparison of Transcriptional and Blood Cell-Phenotypic Markers Between Operationally Tolerant Liver and Kidney Recipients.* American Journal of Transplantation, 2011. **11**(9): p. 1916-1926.

90. McKnight, A.J., D. O'Donoghue, and A. Peter Maxwell, *Annotated chromosome maps for renal disease.* Hum Mutat, 2009. **30**(3): p. 314-20.
