# Supplementary material for: Diagnosis of T-cell-mediated kidney rejection by biopsy-based proteomic biomarkers and machine learning
Source: Front Immunol. 2023 Feb 6;14:1090373. doi: 10.3389/fimmu.2023.1090373 (PMC9939643; doi:10.3389/fimmu.2023.1090373)
Supplement: Supplementary file 2 [file DataSheet_2.docx]

library("e1071")

library(MASS)

library(randomForest)

library(limma)

library(Biobase)

library(readxl)

library(caret)

library(GEOquery)

library(MASS)

#imputation

## Data imputation function

impute_data = function(df, width = 0.3, downshift = 1.8) {

# df = data frame containing filtered

# Assumes missing data (in df) follows a narrowed and downshifted normal distribution

col_names = colnames(df)

# Imputation

set.seed(1)

df[col_names] = lapply(col_names,

function(x) {

temp = df[[x]]

#temp[!is.finite(temp)] = NA

temp.sd = width * sd(temp, na.rm = TRUE) # shrink sd width

temp.mean = mean(temp, na.rm = TRUE) -

downshift * sd(temp, na.rm = TRUE) # shift mean of imputed values

n.missing = sum(is.na(temp))

temp[is.na(temp)] = rnorm(n.missing, mean = temp.mean, sd = temp.sd)

return(temp)

})

return(df)

}

#setwd("D:/research/Dr Randhawa/TCMR_TMT_ML_shortgun/data/")

setwd("/Users/pengliu/Dropbox/OneDrive - University of Pittsburgh/research/other/Xiao/TCMR_TMT_ML_shortgun/data/")

#Read in

data<-read_excel("2nd manuscript.xlsx", sheet = "data for PCA")

data_NKID_TCMR<-unique(data[,c(1,3:12)])

data_NKID_BKPyVN<-unique(data[,c(14,16:25)])

#Remove missing uniprot ids

data_NKID_TCMR<-data_NKID_TCMR[-925,]

group<-c(rep("NKID",5),rep("TCMR",5),rep("NKID",5),rep("BKPyVN",5))

#combine three phenotypes into one big matrix

index<-unique(c(data_NKID_TCMR$`C: Sample...1`,data_NKID_BKPyVN$`C: Sample...14`))

data1<-as.data.frame(data_NKID_TCMR[,2:11])

data2<-as.data.frame(data_NKID_BKPyVN[,2:11])

rownames(data1)<-data_NKID_TCMR$`C: Sample...1`

rownames(data2)<-data_NKID_BKPyVN$`C: Sample...14`

data_all<-cbind(data1[index,],data2[index,])

data_all<-apply(data_all,2,function(x) as.numeric(x))

rownames(data_all)<-index

colnames(data_all)<-c(paste0("NKID_",1:5),paste0("TCMR_",1:5),paste0("NKID_",1:5),paste0("BKPyVN_",1:5))

boxplot(data_all)

#quantile normalization

library(preprocessCore)

data_all<-normalize.quantiles(as.matrix(data_all))

boxplot(data_all)

rownames(data_all)<-index

colnames(data_all)<-c(paste0("NKID_",1:5),paste0("TCMR_",1:5),paste0("NKID2_",1:5),paste0("BKPyVN_",1:5))

## Apply imputation

data_all = impute_data(as.data.frame(data_all))

data_all<-cbind((data_all[,1:5]+data_all[,11:15])/2,data_all[,6:10],data_all[,16:20])

data.corr2<-data_all # change to the same name of data for cv

#---------------------------------------------------------------------------

# Creat common DE list for validation on transcriptome and proteeomic data, we will not use the commonDE list for the cross validation below

setwd("/Users/pengliu/Dropbox/OneDrive - University of Pittsburgh/research/other/Xiao/TCMR_TMT_ML_shortgun/Manuscript/")

CommonDE<-read.table("DE protein panel mapped.txt", header=T) # https://www.uniprot.org/uploadlists/

DElist<-CommonDE[CommonDE$Accession %in% rownames(data_all),]

colnames(DElist)<-c("Uniprot","Genes")

data.train<-data_all[DElist$Uniprot,]

boxplot(data.train)

# PCA plot

library(ggfortify)

label=c(rep("NKID",5),rep("TCMR",5),rep("BKPyVN",5))

df.c.a<-data.frame(condition=label,t(data_all))

autoplot(prcomp(df.c.a[,-1]), data=df.c.a, colour="condition", frame=T)

model=randomForest(x=t(data.train),y=factor(c(1,1,1,1,1,2,2,2,2,2,3,3,3,3,3)))

write.csv(data.frame(features=rownames(model$importance),model$importance)[order(model$importance,decreasing = T),], file="importance_rf.csv")

##-----------------------------------

# cross validation

##-----------------------------------

TMT.DE1<-read_excel("/Users/pengliu/Dropbox/OneDrive - University of Pittsburgh/research/other/Xiao/TCMR_TMT_ML_shortgun/old/manuscript/manuscript05042020/machine learning-supplementary excel tables S1-S12_04182020.xlsx", sheet="Table S5",skip=8) #TCMR vs STA

TMT.DE2<-read_excel("/Users/pengliu/Dropbox/OneDrive - University of Pittsburgh/research/other/Xiao/TCMR_TMT_ML_shortgun/old/manuscript/manuscript05042020/machine learning-supplementary excel tables S1-S12_04182020.xlsx", sheet="Table S6",skip=8) #BKPyVN vs STA

TMT.DE3<-read_excel("/Users/pengliu/Dropbox/OneDrive - University of Pittsburgh/research/other/Xiao/TCMR_TMT_ML_shortgun/old/manuscript/manuscript05042020/machine learning-supplementary excel tables S1-S12_04182020.xlsx", sheet="Table S7",skip=8) #TCMR vs BKPyVN

TMT.DEList1<-TMT.DE1$Accession

TMT.DEList2<-TMT.DE2$Accession

TMT.DEList3<-TMT.DE3$Accession

TMT.DEList<-unique(c(TMT.DEList1,TMT.DEList2,TMT.DEList3)) #TMT DEList

## loocv

flds <- createFolds(1:ncol(data.corr2), k = ncol(data.corr2), list = TRUE, returnTrain = FALSE)

SVM.predict<-numeric(length=ncol(data.corr2))

SVM.prob<-matrix(nrow=ncol(data.corr2),ncol=3)

for(i in 1:length(flds)){

#* DE analysis*#

data.corr2.loo<-data.corr2[,-flds[[i]]]

#Creat a ExpressionSet object

data.corr2.exprs=as.matrix(data.corr2.loo)

eset <- ExpressionSet(assayData=data.corr2.exprs)

#Design matrix

design <- model.matrix(~ 0+factor(c(1,1,1,1,1,2,2,2,2,2,3,3,3,3,3)[-flds[[i]]]))

colnames(design) <- c("TCMR","STA","BKPyVN")

#DE analysis

fit <- lmFit(eset, design)

contrast.matrix <- makeContrasts(TCMR-STA,BKPyVN-STA,TCMR-BKPyVN, levels=design)

fit2 <- contrasts.fit(fit, contrast.matrix)

fit2 <- eBayes(fit2)

res.loo<-topTable(fit2, coef=1:3, adjust="BH", number=nrow(data.corr2.loo))

LF.DElist.loo<- rownames(res.loo)[res.loo$adj.P.Val<0.05] # top genes

common.DElist<-intersect(TMT.DEList,LF.DElist.loo)

#LOOCV

data.corr2.train<- data.corr2[common.DElist, -flds[[i]]]

data.corr2.test<- data.corr2[common.DElist, flds[[i]],drop=F]

temp.traindata<-cbind(class=factor(c(1,1,1,1,1,2,2,2,2,2,3,3,3,3,3)[-flds[[i]]]), data.frame(t(data.corr2.train)))

temp.testdata<-data.frame(t(data.corr2.test))

model<-svm(class ~ ., temp.traindata, probability = T)

SVM.predict[flds[[i]]]<-predict(model, temp.testdata)

SVM.prob[flds[[i]],]<-attributes(predict(model, temp.testdata,probability = T))$probabilities

}

SVM.predict

SVM.prob #https://mmuratarat.github.io/2019-10-12/probabilistic-output-of-svm uses Platt Scaling as in LIBSVM

RF.predict<-numeric(length=ncol(data.corr2))

RF.prob<-matrix(nrow=ncol(data.corr2),ncol=3)

for(i in 1:length(flds)){

#* DE analysis*#

data.corr2.loo<-data.corr2[,-flds[[i]]]

#Creat a ExpressionSet object

data.corr2.exprs=as.matrix(data.corr2.loo)

eset <- ExpressionSet(assayData=data.corr2.exprs)

#Design matrix

design <- model.matrix(~ 0+factor(c(1,1,1,1,1,2,2,2,2,2,3,3,3,3,3)[-flds[[i]]]))

colnames(design) <- c("TCMR","STA","BKPyVN")

#DE analysis

fit <- lmFit(eset, design)

contrast.matrix <- makeContrasts(TCMR-STA,BKPyVN-STA,TCMR-BKPyVN, levels=design)

fit2 <- contrasts.fit(fit, contrast.matrix)

fit2 <- eBayes(fit2)

res.loo<-topTable(fit2, coef=1:3, adjust="BH", number=nrow(data.corr2.loo))

LF.DElist.loo<- rownames(res.loo)[res.loo$adj.P.Val<0.05] # top genes

common.DElist<-intersect(TMT.DEList,LF.DElist.loo)

#LOOCV

data.corr2.train<- data.corr2[common.DElist, -flds[[i]]]

data.corr2.test<- data.corr2[common.DElist, flds[[i]],drop=F]

temp.traindata<- data.frame(t(data.corr2.train))

temp.testdata<-data.frame(t(data.corr2.test))

model<-randomForest(x=temp.traindata,y=factor(c(1,1,1,1,1,2,2,2,2,2,3,3,3,3,3)[-flds[[i]]]))

RF.predict[flds[[i]]]<-predict(model, temp.testdata)

RF.prob[flds[[i]],]<-predict(model, temp.testdata, type="prob")

}

RF.predict

RF.prob

LDA.predict<-numeric(length=ncol(data.corr2))

LDA.prob<-matrix(nrow=ncol(data.corr2),ncol=3)

for(i in 1:length(flds)){

#* DE analysis*#

data.corr2.loo<-data.corr2[,-flds[[i]]]

#Creat a ExpressionSet object

data.corr2.exprs=as.matrix(data.corr2.loo)

eset <- ExpressionSet(assayData=data.corr2.exprs)

#Design matrix

design <- model.matrix(~ 0+factor(c(1,1,1,1,1,2,2,2,2,2,3,3,3,3,3)[-flds[[i]]]))

colnames(design) <- c("TCMR","STA","BKPyVN")

#DE analysis

fit <- lmFit(eset, design)

contrast.matrix <- makeContrasts(TCMR-STA,BKPyVN-STA,TCMR-BKPyVN, levels=design)

fit2 <- contrasts.fit(fit, contrast.matrix)

fit2 <- eBayes(fit2)

res.loo<-topTable(fit2, coef=1:3, adjust="BH", number=nrow(data.corr2.loo))

LF.DElist.loo<- rownames(res.loo)[res.loo$adj.P.Val<0.05] # top genes

common.DElist<-intersect(TMT.DEList,LF.DElist.loo)

#LOOCV

data.corr2.train<- data.corr2[common.DElist, -flds[[i]]]

data.corr2.test<- data.corr2[common.DElist, flds[[i]],drop=F]

temp.traindata<-cbind(class=factor(c(1,1,1,1,1,2,2,2,2,2,3,3,3,3,3)[-flds[[i]]]), data.frame(t(data.corr2.train)))

temp.testdata<-data.frame(t(data.corr2.test))

model<-lda(class ~ ., temp.traindata)

LDA.predict[flds[[i]]]<-predict(model, temp.testdata)$class

LDA.prob[flds[[i]],]<-predict(model, temp.testdata)$posterior

}

LDA.predict

LDA.prob

#scatter plot of LOOCV result --------------------------

layout(matrix(c(1,3,5,2,4,6),ncol=2),height=c(1,1,1),width=c(4,1))

par(mar=c(5,5,3,0))

plot(1:nrow(SVM.prob),SVM.prob[,1],ylim=c(0,1),xlim=c(1,15),col="green",pch=16, xaxt="n",xlab="",ylab="Probability",main="SVM")

abline(v = 5.5, col="black", lty=2)

abline(v = 10.5, col="black", lty=2)

axis(1, at=1:15,labels=colnames(data.corr2), las=2)

par(new=T)

plot(1:nrow(SVM.prob),SVM.prob[,2],ylim=c(0,1),xlim=c(1,15),col="red",axes=FALSE,xlab="",ylab="",pch=16)

par(new=T)

plot(1:nrow(SVM.prob),SVM.prob[,3],ylim=c(0,1),xlim=c(1,15),col="blue",axes=FALSE,xlab="",ylab="",pch=16)

plot.new()

legend("center", c("TCMR","STA","BKPyVN"),pch=c(16,16,16),col=c("green","red","blue"),)

par(mar=c(5,5,3,0))

plot(1:nrow(RF.prob),RF.prob[,1],ylim=c(0,1),xlim=c(1,15),col="green",pch=16,xaxt="n",xlab="",ylab="Probability",main="RF")

abline(v = 5.5, col="black", lty=2)

abline(v = 10.5, col="black", lty=2)

axis(1, at=1:15,labels=colnames(data.corr2), las=2)

par(new=T)

plot(1:nrow(RF.prob),RF.prob[,2],ylim=c(0,1),xlim=c(1,15),col="red",axes=FALSE,xlab="",ylab="",pch=16)

par(new=T)

plot(1:nrow(RF.prob),RF.prob[,3],ylim=c(0,1),xlim=c(1,15),col="blue",axes=FALSE,xlab="",ylab="",pch=16)

plot.new()

legend("center", c("TCMR","STA","BKPyVN"),pch=c(16,16,16),col=c("green","red","blue"),)

par(mar=c(5,5,3,0))

plot(1:nrow(LDA.prob),LDA.prob[,1],ylim=c(0,1),xlim=c(1,15),col="green",pch=16,xaxt="n",xlab="",ylab="Probability",main="LDA")

abline(v = 5.5, col="black", lty=2)

abline(v = 10.5, col="black", lty=2)

axis(1, at=1:15,labels=colnames(data.corr2), las=2)

par(new=T)

plot(1:nrow(LDA.prob),LDA.prob[,2],ylim=c(0,1),xlim=c(1,15),col="red",axes=FALSE,xlab="",ylab="",pch=16)

par(new=T)

plot(1:nrow(LDA.prob),LDA.prob[,3],ylim=c(0,1),xlim=c(1,15),col="blue",axes=FALSE,xlab="",ylab="",pch=16)

plot.new()

legend("center", c("TCMR","STA","BKPyVN"),pch=c(16,16,16),col=c("green","red","blue"),)

#ROC pdf --------------------------

setwd("D:/research/Dr Randhawa/TCMR_TMT_ML_shortgun/ML")

library(precrec)

pdf(file="ROC_TCMRvsSTA_LDA.pdf")

precrec_obj <- evalmod(scores = LDA.prob[10:1,1], labels = c(1,1,1,1,1,2,2,2,2,2))

autoplot(precrec_obj, curvetype = c("ROC"))

dev.off()

pdf(file="ROC_BKPyVNvsSTA_LDA.pdf")

precrec_obj <- evalmod(scores = LDA.prob[c(6:15),3], labels = c(1,1,1,1,1,2,2,2,2,2))

autoplot(precrec_obj, curvetype = c("ROC"))

dev.off()

pdf(file="ROC_TCMRvsBKPyVN_LDA.pdf")

precrec_obj <- evalmod(scores = LDA.prob[c(11:15,1:5),1], labels = c(1,1,1,1,1,2,2,2,2,2))

autoplot(precrec_obj, curvetype = c("ROC"))

dev.off()

pdf(file="ROC_TCMRvsSTA_RF.pdf")

precrec_obj <- evalmod(scores = RF.prob[10:1,1], labels = c(1,1,1,1,1,2,2,2,2,2))

autoplot(precrec_obj, curvetype = c("ROC"))

dev.off()

pdf(file="ROC_BKPyVNvsSTA_RF.pdf")

precrec_obj <- evalmod(scores = RF.prob[c(6:15),3], labels = c(1,1,1,1,1,2,2,2,2,2))

autoplot(precrec_obj, curvetype = c("ROC"))

dev.off()

pdf(file="ROC_TCMRvsBKPyVN_RF.pdf")

precrec_obj <- evalmod(scores = RF.prob[c(11:15,1:5),1], labels = c(1,1,1,1,1,2,2,2,2,2))

autoplot(precrec_obj, curvetype = c("ROC"))

dev.off()

pdf(file="ROC_TCMRvsSTA_SVM.pdf")

precrec_obj <- evalmod(scores = SVM.prob[10:1,1], labels = c(1,1,1,1,1,2,2,2,2,2))

autoplot(precrec_obj, curvetype = c("ROC"))

dev.off()

pdf(file="ROC_BKPyVNvsSTA_SVM.pdf")

precrec_obj <- evalmod(scores = SVM.prob[c(6:15),3], labels = c(1,1,1,1,1,2,2,2,2,2))

autoplot(precrec_obj, curvetype = c("ROC"))

dev.off()

pdf(file="ROC_TCMRvsBKPyVN_SVM.pdf")

precrec_obj <- evalmod(scores = SVM.prob[c(11:15,1:5),1], labels = c(1,1,1,1,1,2,2,2,2,2))

autoplot(precrec_obj, curvetype = c("ROC"))

dev.off()

#ROC tiff -----------------------------

library(precrec)

tiff(file="ROC_TCMRvsSTA_LDA.tiff")

precrec_obj <- evalmod(scores = LDA.prob[10:1,1], labels = c(1,1,1,1,1,2,2,2,2,2))

autoplot(precrec_obj, curvetype = c("ROC"))

dev.off()

tiff(file="ROC_BKPyVNvsSTA_LDA.tiff")

precrec_obj <- evalmod(scores = LDA.prob[c(6:15),3], labels = c(1,1,1,1,1,2,2,2,2,2))

autoplot(precrec_obj, curvetype = c("ROC"))

dev.off()

tiff(file="ROC_TCMRvsBKPyVN_LDA.tiff")

precrec_obj <- evalmod(scores = LDA.prob[c(11:15,1:5),1], labels = c(1,1,1,1,1,2,2,2,2,2))

autoplot(precrec_obj, curvetype = c("ROC"))

dev.off()

tiff(file="ROC_TCMRvsSTA_RF.tiff")

precrec_obj <- evalmod(scores = RF.prob[10:1,1], labels = c(1,1,1,1,1,2,2,2,2,2))

autoplot(precrec_obj, curvetype = c("ROC"))

dev.off()

tiff(file="ROC_BKPyVNvsSTA_RF.tiff")

precrec_obj <- evalmod(scores = RF.prob[c(6:15),3], labels = c(1,1,1,1,1,2,2,2,2,2))

autoplot(precrec_obj, curvetype = c("ROC"))

dev.off()

tiff(file="ROC_TCMRvsBKPyVN_RF.tiff")

precrec_obj <- evalmod(scores = RF.prob[c(11:15,1:5),1], labels = c(1,1,1,1,1,2,2,2,2,2))

autoplot(precrec_obj, curvetype = c("ROC"))

dev.off()

tiff(file="ROC_TCMRvsSTA_SVM.tiff")

precrec_obj <- evalmod(scores = SVM.prob[10:1,1], labels = c(1,1,1,1,1,2,2,2,2,2))

autoplot(precrec_obj, curvetype = c("ROC"))

dev.off()

tiff(file="ROC_BKPyVNvsSTA_SVM.tiff")

precrec_obj <- evalmod(scores = SVM.prob[c(6:15),3], labels = c(1,1,1,1,1,2,2,2,2,2))

autoplot(precrec_obj, curvetype = c("ROC"))

dev.off()

tiff(file="ROC_TCMRvsBKPyVN_SVM.tiff")

precrec_obj <- evalmod(scores = SVM.prob[c(11:15,1:5),1], labels = c(1,1,1,1,1,2,2,2,2,2))

autoplot(precrec_obj, curvetype = c("ROC"))

dev.off()

###############################################################

#Transcriptome dataset GSE48581 for testing

################################################################

# load series and platform data from GEO

gset <- getGEO("GSE48581", GSEMatrix =TRUE, getGPL=FALSE)

if (length(gset) > 1) idx <- grep("GPL570", attr(gset, "names")) else idx <- 1

gset <- gset[[idx]]

# make proper column names to match toptable

fvarLabels(gset) <- make.names(fvarLabels(gset))

# Extract the gene expression

ex2 <- Biobase::exprs(gset)

# use biomart to transfer the probes to gene name

probeids<-rownames(ex2)

library("biomaRt")

ensembl=useMart("ensembl")

ensembl = useDataset("hsapiens_gene_ensembl",mart=ensembl)

re<-getBM(attributes = c('affy_hg_u133_plus_2','hgnc_symbol'),

filters = 'affy_hg_u133_plus_2',

values = probeids,

mart = ensembl)

#remove duplicates

re<-re[re$hgnc_symbol!="",]

ex3<-ex2[re$affy_hg_u133_plus_2,]

id<-re$hgnc_symbol

iqr.range<-apply(ex3,1,IQR)

ex3df<-data.frame(ex3)

library(dplyr)

ex3df<-ex3df %>%

mutate(id=id,iqr.range=iqr.range) %>%

group_by(id) %>%

filter(iqr.range==max(iqr.range)) %>%

unique()

geneNames<-ex3df$id

ex4<-as.matrix(ex3df[,-c(307,308)])

rownames(ex4)<-geneNames

dim(ex4) #21545 x 306

# Select TCMR and STA data

pheno.data<-pData(phenoData(gset))

gsms<-pheno.data$`diagnosis (tcmr, abmr, mixed, non-rejecting, nephrectomy):ch1`

library(plyr)

sml<-mapvalues(gsms,c("non-rejecting","TCMR","mixed","nephrectomy","ABMR"),c("0","1","2","3","4"))

STA.tr<-ex4[,sml==0]

TCMR.tr<-ex4[,sml==1]

data1.test<-cbind(STA.tr,TCMR.tr)

dim(data1.test) #22891 x 254

# 222 STA 32 TCMR

label<-c(rep(1,222),rep(2,32))

##---------------

#Preprocess train and test data

##---------------

#standarize train and test

data.train.sub<-data.train[,1:10]

data.train.sub<-t(apply(data.train.sub,1,function(x) (x-mean(x))/sd(x)))

data1.test<-t(apply(data1.test,1,function(x) (x-mean(x))/sd(x)))

#------------------

# Model fitting and testing

#------------------

genenames<-c()

for(i in 1:length(DElist$Genes)){

temp<-intersect(strsplit(DElist$Genes[i]," ")[[1]],rownames(data1.test))

genenames[i]<-temp[1]

}

data.train.sub<-data.train.sub[!is.na(genenames),]

rownames(data.train.sub)<-genenames[!is.na(genenames)]

#svm

temp.traindata<-cbind(class=factor(c(1,1,1,1,1,2,2,2,2,2)), data.frame(t(data.train.sub)))

temp.testdata<-data.frame(t(data1.test))

model<-svm(class ~ ., temp.traindata)

svm.predict<-predict(model, temp.testdata)

table(svm.predict,label)

# label

#svm.predict 1 2

# 1 142 8

# 2 80 24

#RF

model=randomForest(x=t(data.train.sub),y=factor(c(1,1,1,1,1,2,2,2,2,2)))

rf.predict=predict(model,t(data1.test))

table(rf.predict,label)

# label

#rf.predict 1 2

# 1 143 7

# 2 79 25

write.csv(model$importance, file="importance_rf_GSE48581.csv")

#LDA

temp.traindata<-cbind(class=factor(c(1,1,1,1,1,2,2,2,2,2)), data.frame(t(data.train.sub)))

temp.testdata<-data.frame(t(data1.test))

model<-lda(class ~ ., temp.traindata)

lda.predict<-predict(model, temp.testdata)$class

table(lda.predict, label)

# label

#lda.predict 1 2

# 1 136 8

# 2 86 24

###############################################################

#Transcriptome dataset GSE36059 for testing

################################################################

# Differential expression analysis with limma

library(Biobase)

library(GEOquery)

library(limma)

library(MASS)

# load series and platform data from GEO

gset2 <- getGEO("GSE36059", GSEMatrix =TRUE, getGPL=FALSE)

if (length(gset2) > 1) idx <- grep("GPL570", attr(gset22, "names")) else idx <- 1

gset2 <- gset2[[idx]]

# make proper column names to match toptable

fvarLabels(gset2) <- make.names(fvarLabels(gset2))

ex2 <- Biobase::exprs(gset2)

# use biomart to transfer the probes to gene name

probeids<-rownames(ex2)

library("biomaRt")

ensembl=useMart("ensembl")

ensembl = useDataset("hsapiens_gene_ensembl",mart=ensembl)

re<-getBM(attributes = c('affy_hg_u133_plus_2','hgnc_symbol'),

filters = 'affy_hg_u133_plus_2',

values = probeids,

mart = ensembl)

#remove duplicates

re<-re[re$hgnc_symbol!="",]

ex3<-ex2[re$affy_hg_u133_plus_2,]

id<-re$hgnc_symbol

iqr.range<-apply(ex3,1,IQR)

ex3df<-data.frame(ex3)

library(dplyr)

ex3df<-ex3df %>%

mutate(id=id,iqr.range=iqr.range) %>%

group_by(id) %>%

filter(iqr.range==max(iqr.range)) %>%

unique()

geneNames<-ex3df$id

ex4<-as.matrix(ex3df[,-c(412,413)])

rownames(ex4)<-geneNames

dim(ex4) #21545 x 411

# Select TCMR and STA data

library(readxl)

GSE36059<-read_excel("D:/research/Dr Randhawa/TCMR vs STA paper/my analysis/GSE36059.xlsx",col_names = F)

colnames(GSE36059)<-c("X__1","X__2")

GSE36059$X__2[grep("non-rejecting",GSE36059$X__2)]<-0

GSE36059$X__2[grep("TCMR",GSE36059$X__2)]<-1

GSE36059$X__2[grep("MIXED",GSE36059$X__2)]<-2

GSE36059$X__2[grep("Nephrectomy",GSE36059$X__2)]<-3

GSE36059$X__2[grep("ABMR",GSE36059$X__2)]<-4

STA.tr<-ex4[,GSE36059[GSE36059$X__2 == 0,]$X__1]

TCMR.tr<-ex4[,GSE36059[GSE36059$X__2 == 1,]$X__1]

data2.test<-cbind(STA.tr,TCMR.tr)

dim(data2.test) #21835 316

#281 sta 35 TCMR

label<-c(rep(1,281),rep(2,35))

save(data1.test,data2.test,file="/Users/pengliu/Dropbox/OneDrive - University of Pittsburgh/research/other/Xiao/TCMR_TMT_ML_shortgun/ML/Test_transcriptome.rdata")

##---------------

#Proprocess train and test data

##---------------

#standarize train and test

data.train.sub<-data.train[,1:10]

data.train.sub<-t(apply(data.train.sub,1,function(x) (x-mean(x))/sd(x)))

data2.test<-t(apply(data2.test,1,function(x) (x-mean(x))/sd(x)))

#------------------

# Model fitting and testing

#------------------

genenames<-c()

for(i in 1:length(DElist$Genes)){

temp<-intersect(strsplit(DElist$Genes[i]," ")[[1]],rownames(data2.test))

#print(length(temp))

genenames[i]<-temp[1]

}

data.train.sub<-data.train.sub[!is.na(genenames),]

rownames(data.train.sub)<-genenames[!is.na(genenames)]

#svm

temp.traindata<-cbind(class=factor(c(1,1,1,1,1,2,2,2,2,2)), data.frame(t(data.train.sub)))

temp.testdata<-data.frame(t(data2.test))

model<-svm(class ~ ., temp.traindata)

svm.predict<-predict(model, temp.testdata)

table(svm.predict,label)

# label

#svm.predict 1 2

# 1 170 9

# 2 111 26

#RF

model=randomForest(x=t(data.train.sub),y=factor(c(1,1,1,1,1,2,2,2,2,2)))

rf.predict=predict(model,t(data2.test))

table(rf.predict,label)

# label

#rf.predict 1 2

# 1 165 6

# 2 116 29

write.csv(model$importance, file="importance_rf_GSE36059.csv")

#LDA

temp.traindata<-cbind(class=factor(c(1,1,1,1,1,2,2,2,2,2)), data.frame(t(data.train.sub)))

temp.testdata<-data.frame(t(data2.test))

model<-lda(class ~ ., temp.traindata)

lda.predict<-predict(model, temp.testdata)$class

table(lda.predict,label)

# label

#lda.predict 1 2

# 1 182 10

# 2 99 25

###############################################################

# Independent biopsies for validation

################################################################

#---------------------------------

# Proteomic data for Validation

#---------------------------------

setwd("D:/research/Dr Randhawa/TCMR_TMT_ML_shortgun/data")

data_valid<-read_excel("verified data-2.xlsx", sheet = "with normalize & impute")

data_valid<-as.data.frame(data_valid)

data_valid<-data_valid[,-seq(3,29,3)]

index.test<-unique(c(data_valid$Accession...1, data_valid$Accession...4,

data_valid$Accession...7, data_valid$Accession...10,

data_valid$Accession...13, data_valid$Accession...16,

data_valid$Accession...19, data_valid$Accession...22,

data_valid$Accession...25, data_valid$Accession...28))

index.test<-index.test[-which(is.na(index.test))]

data_valid2<-matrix(nrow=length(index.test),ncol=10)

rownames(data_valid2)<-index.test

colnames(data_valid2)<-colnames(data_valid[,seq(2,20,2)])

for(i in 1:10){

temp<-data_valid[,c(2*i-1, 2*i)]

temp<-temp[!is.na(temp[,1]),]

rownames(temp)<-temp[,1]

temp<-temp[,-1,drop=F]

data_valid2[,i]<-temp[index.test,]

}

data_valid3<-data_valid2[apply(data_valid2,1,function(x) sum(is.na(x)))<5,]

data_valid3

#quantile normalization

library(preprocessCore)

data_valid4<-normalize.quantiles(as.matrix(data_valid3))

rownames(data_valid4)<-rownames(data_valid3)

colnames(data_valid4)<-colnames(data_valid3)

## Apply imputation

data_valid5 = impute_data(as.data.frame(data_valid4))

#normalize train and test

data.train.sub<-data.train[,1:10]

#data.train.sub<-t(apply(data.train.sub,1,function(x) (x-mean(x))/sd(x)))

data2.test<-data_valid5

#boxplot(data2.test)

#quantile normalize the training and testing data together

index<-intersect(rownames(data.corr2), rownames(data2.test))

data.combined<-cbind(data.corr2[index,1:10], data2.test[index,])

data.combined.norm<-normalize.quantiles(as.matrix(data.combined))

colnames(data.combined.norm)<-colnames(data.combined)

rownames(data.combined.norm)<-rownames(data.combined)

#subset the genes in the DElist, and prepare the data for model fitting

data.train.sub<-data.combined.norm[intersect(DElist$Uniprot, rownames(data2.test)),1:10]

data2.test<-data.combined.norm[intersect(DElist$Uniprot, rownames(data2.test)),11:20]

#rearrange the columns of data2.test

data2.test<-data2.test[,c("PHS18_28595_cortex","PHS18_19976","PHS18_22368","PHS18_25950","PHS18_26454","PHS17_27724_cortex","PHS17_27724_medulla","PHS18_896","PHS18_22866","PHS18_2207")]

label<-c(rep("NKID",5),rep("TCMR",5))

library(ggfortify)

label=c(rep("NKID",5),rep("TCMR",5))

df.c.a<-data.frame(condition=label,t(data2.test))

autoplot(prcomp(df.c.a[,-1]), data=df.c.a, colour="condition", frame=T)

#------------------

# Model fitting and testing

#------------------

#svm

temp.traindata<-cbind(class=factor(c(1,1,1,1,1,2,2,2,2,2)), data.frame(t(data.train.sub)))

temp.testdata<-data.frame(t(data2.test))

model<-svm(class ~ ., temp.traindata)

svm.predict<-predict(model, temp.testdata)

#plot(attributes(svm.predict)$probabilities[,1])

table(svm.predict,label)

# label

# svm.predict NKID TCMR

# 1 2 1

# 2 3 4

#RF

model=randomForest(x=t(data.train.sub),y=factor(c(1,1,1,1,1,2,2,2,2,2)), ntree = 10000)

rf.predict=predict(model,t(data2.test))

table(rf.predict,label)

# label

# rf.predict NKID TCMR

# 1 5 1

# 2 0 4

#LDA

temp.traindata<-cbind(class=factor(c(1,1,1,1,1,2,2,2,2,2)), data.frame(t(data.train.sub)))

temp.testdata<-data.frame(t(data2.test))

model<-lda(class ~ ., temp.traindata)

lda.predict<-predict(model, temp.testdata)

plot(lda.predict$posterior[,1])

table(lda.predict$class,label)

# label

# svm.predict NKID TCMR

# 1 5 2

# 2 0 3
